# A systematic review and meta-analysis of traditional insect Chinese medicines combined chemotherapy for non-surgical hepatocellular carcinoma therapy

**DOI:** 10.1038/s41598-017-04351-y

**Published:** 2017-06-28

**Authors:** Zhaofeng Shi, Tiebing Song, Yi Wan, Juan Xie, Yiquan Yan, Kekai Shi, Yongping Du, Lei Shang

**Affiliations:** 1 Department of Traditional Chinese Medicine, Xijing Hospital Affiliated to Fourth Military Medical University, Xi’an, 710032 China; 2 Department of Orthopaedics, Xi’an City Hospital of Traditional Chinese Medicine, Xi’an, 710021 China; 3 Department of Health Services, the Public Health Faculty of Fourth Military Medical University, Xi’an, 710032 China; 4 Department of Health Statistics, the Public Health Faculty of Fourth Military Medical University, Xi’an, 710032 China; 5 Department of Engineering, Carnegie Mellon University, Pittsburgh, PA15213 United States

## Abstract

On the background of high morbidity and mortality of hepatocellular carcinoma (HCC) and rapid development of traditional Chinese medicine (TCM), we conducted a systematic review and meta-analysis of randomized clinical trials (RCTs) according to the Preferred Reporting Items for Systematic Reviews and Meta-Analyses (PRISMA) statement to assess the clinical effectiveness and safety of traditional insect Chinese medicine and related preparation for non-surgical HCC. RCTs were searched based on standardized searching rules in mainstream medical databases from the inception up to May 2016. Ultimately, a total of 57 articles with 4,651 patients enrolled in this meta-analysis. We found that traditional insect Chinese medicine and related preparation combined chemotherapy show significantly effectiveness and safety in objective response rate (*P* < 0.001), survival time extension [12 months (*P* < 0.001); 18 months (*P* < 0.001); 24 months (*P* < 0.001); 36 months (*P* < 0.001)], amelioration for life quality [QoL scores improvement (*P* < 0.001); KPS improvement (*P* < 0.001); AFP improvement (*P* < 0.001)] and reduction of therapeutic toxicity [WBC decrease (*P* = 0.04); gastrointestinal adverse reactions (*P* < 0.001)]. In conclusion, traditional insect Chinese medicine and related preparations could be recommended as auxiliary therapy combined chemotherapy for HCC therapy.

## Introduction

Hepatocellular carcinoma (HCC), a common kind of primary liver cancer (PLC), ranks as the sixth most common neoplasm and the third most frequent reason for cancer death^1^. A report by the World Health Organization (WHO) showed that HCC has become a major health problem nowadays, and the incidence of HCC is increasing as time goes on^2^. It was estimated that 748,300 new liver cancer cases and 695,900 liver cancer deaths occurred worldwide in 2008^3^. The morbidity and mortality of HCC reached a new peak in 2012 with 782,000 new cases and 745,000 death cases all over the world^4^. The East and South-East Asia along with the Middle and Western Africa have the highest HCC rates compared with other places around the world^5^. Moreover, the rate of HCC in developed countries, such as the United States, has increased rapidly in recent years^6^.

HCC is the major cause of death among patients who had been diagnosed with cirrhosis^7^. It is estimated that the combined effectiveness of the chronic hepatitis C virus (HCV) and the hepatitis B virus (HBV) infections takes up over 80% of liver cancer cases around the world^8^. With respect to HCC diagnosis, if tumor (lesions ≥ 10mm) shows the typical feature on computed tomography (CT) or magnetic resonance images (MRI), no further investigation will be required. If not, the biopsy specimen is required^9^. The measurement of alpha-fetoprotein (AFP) may not be useful in clinical practice and will not affect the formulation of final treatment strategy^10^.

Although HCC has a poor prognosis so far, ultrasonography surveillance can diagnose HCC at early stage (single tumors or as many as 3 nodules ≤ 3 cm) when the tumor could be cured by resection, liver transplantation, or ablation, and the 5-year survival rate can exceed 50%^10^. For the patients at the intermediate or end stage of HCC, palliative methods such as transcatheter hepatic arterial chemoembolization (TACE) or intravenous chemotherapy are appropriately approaches^11^. Those methods are limited by their toxicities, drug resistance, and miscellaneous adverse effects for end stage patients, meanwhile the survival benefits are still not proven^12^. Given this limitation of therapy on HCC, complementary and alternative medicine (CAM) has been increasingly applied to it over the past few decades. Physicians are trying to find more adjunctive or auxiliary therapies to improve patients’ quality of life or survival time and reduce side effects caused by chemotherapy.

Traditional insect Chinese medicine is an old ancient Chinese medical concept that is still used today in China. It can be recognized as the Chinese animal species medicines, including the dried medical animal bodies, the medical animal bodies without entrails, secretions and excretions of medical animals and the processing products of insects^13^. Owing to the various versions of the translation for TCM, we could not even find a precise translation for traditional insect Chinese medicine from a number of Chinese-English dictionaries of TCM. As a result, we adopted the word “insect” that has the same literary meaning of “animal” to replace it. The traditional insect Chinese medicine is a broad concept, while the narrow definition of it is only confined to the insect or worm drugs. It has been originally recorded in medical book named *Fifty-two patients side* for the medical interpretation and practice in the Warring States period of China (475-221BC)^14^. And *Shennong’s herbal classic*, one of the earliest Chinese herbal collections of TCM, deeply introduced the effects of traditional insect Chinese medicine^15^. Another Chinese herbal masterpiece, *C* *ompendium of materia medica*, written by Shizhen Li, collected nearly 500 kinds of traditional insect Chinese medicines and classified them meticulously, promoting traditional insect Chinese medicine to a new peak. The modern experimental researches of traditional Chinese insect medicine have been gradually focussing on anti-tumor properties by inhibiting the proliferation of cancer cells, inducing tumor cell apoptosis and improving the immune function of the human body^16^.

Traditional insect Chinese medicine, as an essential component of TCM, has been widely applied in the treatment of the non-surgical HCC (at the intermediate stage and the advanced stage) for a long time with side effects a rare occurance. Although a great number of published clinical studies have evaluated different kinds of traditional insect Chinese medicines and the related preparations combined chemotherapy for HCC^17–19^, the clinical efficacy and pharmacological mechanism remain unclear. No article of systematic review or meta-analysis has been conducted based on considering the traditional insect Chinese medicines as an integral part to evaluate the efficiency and safety for HCC treatment. Therefore, we conducted a systematic review and meta-analysis to investigate the evidence about traditional insect Chinese medicine and the related preparation combined chemotherapy for the clinical effectiveness and safety of non-surgical HCC.

## Materials and Methods

This systematic review and meta-analysis was conducted by order of the Preferred Reporting Items for Systematic Reviews and Meta-Analyses (PRISMA) statement^20^.

### Search strategies

Literatures were extracted from the main electronic databases including PubMed, Embase, Medline, Science Direct (SD), Web of Science, ProQuest, the Cochrane Central Register of Controlled Trials, Springerlink, Wiley Library Online, Chinese Biological Medicine Database (CBM), China National Knowledge Infrastructure (CNKI), Chinese Medical Citation Index (CMCI), WanFang Database, Chinese Scientific Journal Database (VIP), and Japan Medical Abstracts Society from their inception up to May 2016. In addition, there were no language restrictions on the literature searching.

The searching terms were performed as follows: “liver cancer,” “hepatocellular carcinoma,” “liver neoplasm,” “primary liver carcinoma,” “traditional insect Chinese medicine,” “insect drugs,” “cinobufatalin,” “huachansu,” “centipede,” “scorpion,” “leech,” “earthworm,” “cantharides,” “gecko,” “snake venom,” “grand beetle,” “aspongopus,” “gadfly,” “honeycomb,” “chemotherapy,” “interventional therapy” and “random clinical trials”. No other restrictions were performed and the free text strategy and Medical Subject Headings (MeSH) terms were conducted in the term searching process. Categories of traditional insect Chinese medicine on literature searching were derived from the classification of MeSH term in CBM database. The searching language in Chinese, English and Japanese was slightly changed based on the situation for different databases adaptation.

### Study selection

The study selection was conducted by two reviewers (Z.F.Shi, Y.Q.Yan) independently and disagreements were resolved by the common strategy or a third reviewer (J.Xie). Eligible studies that suited for the following criteria were included in this systematic review and meta-analysis: (1) randomized clinical trials (RCTs); (2) patients were diagnosed as non-surgical HCC (TNM stage II or above; BCLC stage B or above) by pathological results or imaging examinations; (3) participants received traditional insect Chinese medicine and related preparation combined chemotherapy in the treatment group, meanwhile chemotherapy alone in the control group; (4) evaluated outcomes including at least one of the following variables: (a) complete response rate (CR), partial response rate (PR) and CR + PR as a proportion of objective response rate; (b) quality of life (QoL) scores or Karnofsky performance scores (KPS); (c) the Child-Pugh scores; (d) major side effects resulting from traditional insect Chinese medicines or chemotherapy; (5) survival time rate (the number of participants in the treatment and the control group who were alive at 6, 12, 18, 24, 36 months). The studies were excluded if they met these following criteria: (a) received the Chinese herbal medicine in the control group; (b) received the irrelevant TCM therapies or western medical methods in experimental group; (c) the clinic pathologic types of liver cancer were not compatible with the HCC criteria; (d) severe clinical illnesses (such as liver or kidney disease) or infections; (e) absence or inconsistency of methods, evaluation criteria or results; (f) non-randomized clinical trials (comments, case reports, reviews, editorials, letters, *et al*.), articles unrelated with the topic, or duplicated articles.

Particularly, the clinical stage of HCC was identified according to the TNM staging system of the American Joint Committee on Cancer (AJCC) obtained from the National Comprehensive Cancer Network (NCCN) guideline 2015^21^ (T refers to the size of the original tumor and whether it has invaded nearby tissue; N represents nearby lymph nodes that are involved; M can be recognized as distant metastasis) or the Barcelona Clinical Liver Cancer (BCLC) system coming from the European Association for the Study of the Liver (EASL)^22^. For the reason of diversity and complexity of TCM therapy, traditional insect Chinese medicine and the related preparation can be obtained ﻿from some Chinese herbs that have the synergistic effects without interfering with the major function of traditional insect Chinese medicine. The imaging examination includes the methods of computed tomography (CT) or magnetic resonance imaging (MRI), showing the lesions ≥ 10mm at least. Also, CT or MRI can evaluate the CR or PR: CR means all targeted lesions disappear and pathological lymph nodus must reduce to less than 10mm at least, and PR means targeted lesions decrease 30% in the sum of diameters at least. This solid tumor response was evaluated according to the Response Evaluation Criteria in Solid Tumor (RECIST) guideline (version 1.1), which has been updated and revised by the European Cancer Organization (ECCO) in 2009^23^. The side effects (the chemotherapy related toxicity and TCM adverse effects) included as the follows: bone marrow suppression including leukopenia, thrombocytopenia or anemia; gastrointestinal side effects including nausea, emesis, anorexia, or diarrhea; liver or kidney injury; pyrexia; and pain. The dosage of traditional insect Chinese medicine and chemotherapy in experimental groups and control groups of trials was discrepant. There was no limitation for the dosage in the study searching and including.

### Data extraction and quality analysis

Two reviewers (Z.F.Shi, Y.Q.Yan) extracted data independently, assessing the inclusion and exclusion criteria before based on the standardized collection. All disagreements were discussed between two reviewers mentioned before, and the final conclusion was reached after deliberation over with a third reviewer (J.Xie). The extracted characteristics comprised the following items: (1) the name of author and the year of publication; (2) sample size; (3) study design; (4) study performed areas; (5) the baseline characteristics of patients; (6) approaches and duration of treatments; (7) outcome evaluation and trials quality assessment.

The quality analysis was performed by two investigators independently (Z.F.Shi, Y.Q.Yan), using the Cochrane Collaboration’s tools for assessing the risk of bias^24^. This tool was conducted to evaluate the bias of studies across six domains: (1) the method of random allocation; (2) the concealment of allocation; (3) the blinding method; (4) the integrity of outcome data; (5) the outcome data of selective reports; (6) other bias sources. Every domain mentioned above was assessed by the criterion of “yes”, “no” or “unclear”. The article had 3 or more “yes” could be recognized as high quality and less than 3 “yes” should be considered as low quality. High quality studies had the low bias risk while the low ones had the high bias risk. The related data was analyzed by the software named the Review Manager (RevMan; version 5.2 the Nordic Cochrane Center, the Cochrane Collaboration, 2012 Copenhagen, Denmark), with the outcomes illustrated by images or tables efficiently and conveniently.

### Statistical analysis

The RevMan (version 5.2) was applied to pool and analyze data. The reviewer (Z.F.Shi) calculated the risk ratio (*RR*) with 95% confidence interval (*CI*) for the dichotomous outcomes and the standard mean difference (Std.MD) or mean difference (MD) with 95% confidence interval (*CI*) for the continuous outcomes respectively. Statistical heterogeneity was tested through studies by the method of *I* ^*2*^ statistic. It was a quantitative tool for inconsistency and could provide an estimate of variation resulted from heterogeneity. Results of *I* ^*2*^ statistic between 25 and 50% were regarded as low heterogeneity, 50 and 75% were moderate heterogeneity, and above 75% were high heterogeneity. It should be noted that the Cochrane Handbook (version 5.1.0) indicates the value of *I* ^*2*^ statistic above 50% was considered to have considerable heterogeneity^25^. The subgroup analysis was performed to find the source of heterogeneity. A fixed effects model (applying Mantel-Haenszel method^26^) was conducted to pool data when the heterogeneity did not exist or moderate, while a random effects model (applying Der Simonian-Laired method^27^) was performed when there was obvious heterogeneity. Additionally, sensitivity analysis was further conducted to assess the stability of results and evaluate the variation of pooled data. Publication bias was assessed visually by funnel plots and calculated in Egger’s test/Begg’s test through the software named Stata (version 14.0, StataCrop LP, College Station, US)^28, 29^. *P* values lower than 0.05 were judged as statistically significant for the results, representing that the study has publication bias.

## Results

**Figure 1:**
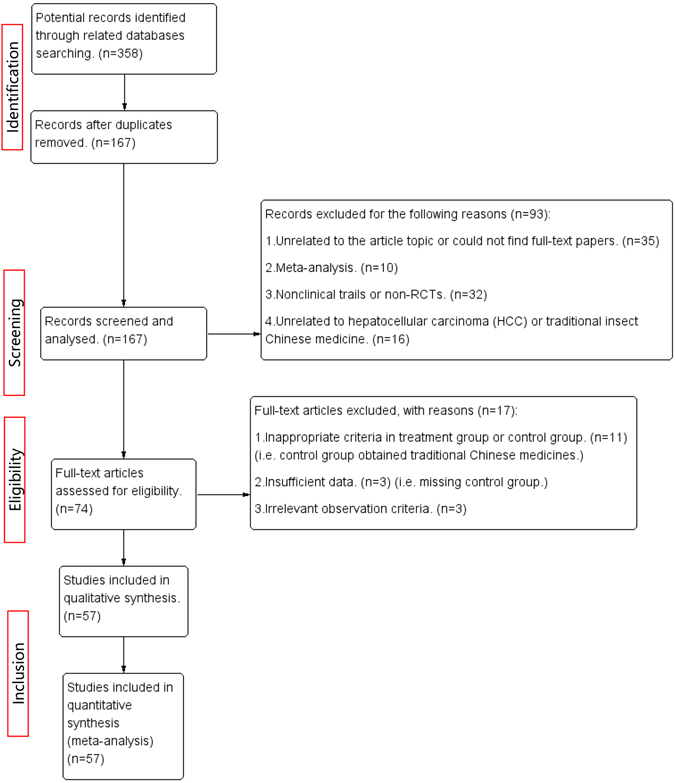
The Preferred Reporting Items for Systematic Reviews and Meta-Analyses (PRISMA) Flow Diagram.

The flowchart of article search and selection in the meta-analysis is presented in Fig. 1. Of a total of 358 potentially relevant articles identified from 15 different electronic databases, one hundred and ninety-one studies of them were ruled out for the reason of duplication. Ninety-three publications were further excluded, after detailed screening and analyzing, for the following reasons: (a) thirty-five studies were ﻿confirmed unrelated to article topics or could not find the full-text papers even after contacting with authors; (b) ten studies were found to be systematic reviews and meta-analyses; (c) Thirty-two studies were non-clinical trials or non-randomized clinical trials; (d) Sixteen articles were unrelated to hepatocellular carcinoma or traditional insect Chinese medicine. Seventy-four full-text articles were eligible and then reviewed independently. Of 17 publications further excluded, 11 publications were excluded because the inappropriate criteria of experimental or control group, for instance, the control group contains TCM therapy. Three publications missed sufficient data (i.e. Control group), which made the report outcomes untrustworthy. Three publications obtained irrelevant observation criteria, which seemed inconsistent with the criteria mentioned before. Overall, a total of 57 articles with 4,651 patients were enrolled in this meta-analysis^30–86^. (Table 1)

**Table 1:** General Characteristics of Included Randomized Clinical Trials

	Experimental group	Control group	Sex(M/F)	Age	Disease stage	Child-Pugh scores	KPS score	Treatment group	Control group		
Huang W.K.^30^ 2013	41	41	44/26	48.54 ± 4.16 years old	II, III	—	>60	Cinobufotalin injection + TACE (OXA, EPI)	TACE (OXA, EPI,)	3 weeks	1. Clinical effectiveness
											2. Life quality
											3. Blood routine examination
Cao Y.^31^ 2014	68	68	78/58	E:55.2 ± 7.4 years old	—	A and B	>50	Compound cantharis capsule + FAP (5-FU, EPI, DDP)	FAP (5-FU, EPI, DDP)	10 days	1. Clinical effectiveness
											2. Life quality
				C:57.8 ± 6.9 years old							3. Complication
											4. Economic evaluation
Zeng C.S.^32^ 2012	30	30	19-Nov	E:51.65 ± 6.92 years old	II, III	A and B	—	Jinlong capsule + TACE (THP, DDP, 5-FU)	TACE (THP, DDP, 5-FU)	60 days	1. Clinical effectiveness
											2. AFP
				C:53.20 ± 9.24 years old							3. Liver function
											4. Complication
											5. Life quality
Deng Z.Y.^33^ 2015	25	24	29/20	E:48.65 ± 16.12 years old	—	—	—	Cinobufotalin injection + TACE (THP, DDP)	TACE (THP, DDP)	4 weeks	1. AFP
											2. Clinical effectiveness
				C:48.30 ± 16.24 years old							3. TCM symptoms scores + KPS
											4. Complication
Dong M.E.^34^ 2014	60	60	76/44	E:median age 55 years	—	—	—	Cinobufotalin injection + TACE (GEM, DDP, 5-FU, CF)	TACE (GEM, DDP, 5-FU, CF)	4 weeks	1. Clinical effectiveness
				C:median age 53 years							2. Complication
Feng X.M.^35^ 2012	38	32	44/26	E:52.6 ± 11.7 years old	II III	—	>60	Cinobufotalin injection + FOLFOX4 (OXA, 5-FU, CF)	FOLFOX4 (OXA, 5-FU, CF)	3 rounds (2 days per round)	1. Clinical effectiveness
				C:53.2 ± 11.4 years old							2. Life quality
											3. Blood routine examination
Fu Z.L.^36^ 2010	78	78	119/37	E:median age 58 years	—	—	>60	Cinobufotalin injection + TACE (5-FU, DDP, MMC)	TACE (5-FU, DDP. MMC)	3 months	1. Clinical effectiveness
				C:median age 56 years							2. Life quality
											3. Complication
He S.L.^37^ 2012	26	25	37/14	58.8 years old	—	A and B	—	Cinobufotalin injection + TACE (OXA, 5-FU, THP)	TACE (OXA, 5-FU, THP)	2 weeks	1. Clinical effectiveness
											2. MST and TTP
											3. VEGF, HIF-1α and AFP
											4. KPS
											5. Complication
Ji J.F.^38^ 2015	25	22	36/11	62.3 years old	—	—	—	Cinobufotalin injection + TACE (DDP, 5-FU, DOX)	TACE (DDP, 5-FU, DOX)	4–5 weeks	1. Clinical effectiveness
											2. Survival at 6/12/24 months
											3. Life quality
											4. Comparison of WBC,TBIL and ALT
											5. Immunological function
Jia C.H.^39^ 2008	30	30	44/16	E:54.2 ± 6.4 years old	I, II, III	A, B and C	>60	Jinlong capsule + TACE (MMC)	TACE (MMC)	3 months	1. Clinical effectiveness
				C:51.5 ± 7.0 years old							2. Immunological function. 3. Life quality
Jiang C.Y.^40^ 2013	30	33	36/27	E:median age:57 years	II, III	—	≥ 60	Jinlong capsule + TACE (5-FU, THP)	TACE (5-FU, THP)	2 rounds (8 days per round)	1. Clinical effectiveness
				C:median age:53 years							2. CBR
											3. Complication
Ke J.^41^ 2011	38	40	69/9	E:58.32 ± 11.59 years old	—	A and B	>60	Cinobufotalin injection + TACE (5-FU, DDP, DOX)	TACE (5-FU, DDP, DOX)	3 rounds (20 days per round)	1. Clinical effectiveness
				C:57.09 ± 11.77 years old							2. Life quality
											3. Comparison of WBC, TBIL, ALT and AFP
											4. Survival at 6/12 months
											5. Complication
Kou C.Y.^42^ 2011	31	31	40/22	E:40.5 years old	—	—	>60	Cinobufotalin injection + TACE (HCPT, DDP, DOX)	TACE (HCPT, DDP, DOX)	4 weeks	1. Clinical effectiveness
				C:41 years old							2. QoL scores
											3. Complication
											4. Survival at 12/24/36 months
Li B.^43^ 2013	74	73	97/50	Median age:56.4 years	—	—	>60	Jinlong capsule + TACE (5-FU, EPI, DDP, MMC, CF)	TACE (5-FU, EPI, DDP, MMC, CF)	2 rounds (28 days per round)	1. Clinical effectiveness
											2. Life quality
											3. Comparison of Child-pugh, Classification and WBC
Li j.^44^ 2013	43	62	76/29	E:45.2 ± 4.8 years old	—	A, B and C	—	Aidi injection + TACE (5-FU, THP, DDP, MMC)	TACE (5-FU, THP, DDP, MMC)	10 days	1. Life quality
				C:45.7 ± 6.4 years old							2. Survival at 6/12/24/36 months
											3. AFP
											4. TACE times
Li Q.^45^ 2008	50	46	84/12	50.2 years old	I, II, III	A and B	≥ 60	Cinobufotalin injection + TACE (5-FU, HCPT, DOX, MMC)	TACE (5-FU, HCPT, DOX, MMC)	4 weeks	1. Clinical effectiveness
											2. Life quality
											3. Survival at 6/12/24 months
											4. Comparison of WBC, TBIL and ALT
											5. Immunological function
Li Q.M.^46^ 2003	20	18	28-Oct	29–65 years old	II, III	—	≥ 60	Qining injection + TACE (HCPT, 5-FU, MMC)	TACE (HCPT, 5-FU, MMC)	7 days	1. Clinical effectiveness
											2. Complication
Li W.H.^47^ 2006	19	19	28-Oct	45 years old	—	—	—	Cinobufotalin injection + TACE (5-FU, DDP, MMC)	TACE (5-FU, DDP, MMC)	2 rounds	1. Life quality
											2. Clinical effectiveness
											3. Comparison of WBC and AFP
											4. Survival at 12/24 months and median survival time
											5. Complication
Liang B.L.^48^ 2010	20	20	30-Oct	E:59 ± 6 years old	—	—	≥ 50	Sodium Cantharidinate and vitamin B6 + TACE (MMC, 5-FU, EPI)	TACE (MMC, 5-FU, EPI)	4weeks	Comparison of liver function and blood routine examination
				C:55 ± 8 years old							
Liang C.X.^49^ 2015	30	30	34/26	E:median age 53 years	—	A and B	>80	Sodium Cantharidinate and Vitamin B6 + TACE (5-FU, DDP, OXA, EPI)	TACE (5-FU, DDP, OXA, EPI)	3–4 rounds (15 days per round)	1. Clinical effectiveness
				C:median age 52 years							2. Comparison of WBC and AFP
											3. QoL scores
Liang.T.J.^50^ 2005	116	108	187/37	E:52.1 ± 9.7 years old	—	A, B and C	—	Jinlong capsule + TACE (EPI, MMC, CBP)	TACE (EPI, MMC, CBP)	≥ 3 years	1. Survival at 6/12/24/36 months
				C:50.4 ± 8.5 years old							2. Clinical effectiveness
											3. QoL scores
Liang Y.^51^ 2008	48	48	72/24	Median age:44.5 years	—	A, B and C	≥ 70	Cinobufotalin injection + TACE (EPI, DDP, 5-FU) and IFN	TACE (EPI, DDP, 5-FU) and IFN	2 rounds (4 weeks)	1. Clinical effectiveness
											2. Live quality
											3. Comparison of 6/12/18 months
											4. Complication
Liu X.H.^52^ 2009	42	42	70/14	48.5 years old	—	—	—	Cinobufotalin injection + TACE (5-FU, DDP, EPI)	TACE (5-FU, DDP, EPI)	2–3 rounds (4 weeks per round)	1. Clinical effectiveness
											2. Survival at 12/24/36 months
											3. Comparison of immune function and liver function
											4. Complication
Liu Y.Q.^53^ 2010	38	44	72/10	E:54.21 ± 10.32 years old	—	A and B	>60	Cinobufotalin injection + TACE (THP, DDP, MMC)	TACE (THP, DDP, MMC)	2–3 rounds (3weeks per round)	1. Lipiodol deposition after TACE
											2. Clinical effectiveness
				C:55.32 ± 11.62 years old							3. Comparison of TTP
											4. Complication
Lu S.J.^54^ 2014	30	30	33/17	E:46.5 years old	—	—	—	Cinobufotalin injection + THM and TACE (DDP, DOX, 5-FU)	TACE (DDP, DOX, 5-FU)	30 days	1. Clinical effectiveness
				C:47.3 years old							2. Life quality
											3. Survival at 6/12/24 months
											4. Complication
Peng W.D.^55^ 2011	40	40	43/37	E:45.0 ± 13.5 years old	II, III	—	≥ 50	Compound cantharis capsule + FAP (5-FU, EPI, DDP)	FAP (5-FU, EPI, DDP)	10 days	1. Clinical effectiveness
				C:44.0 ± 13.9 years old							2. Life quality
											3. Immunological function
Qu J.R.^56^ 2012	40	40	64/16	E:median age 54 years	II, III	—	≥ 60	Secretio bufonis injection + TACE (CBP, 5-FU, MMC,DOX)	TACE (CBP, 5-FU, MMC, DOX)	28 days	1. Complication
				C:median age 56 years							2. Comparison of liver function and blood routine examination
Shen J.J.^57^ 2009	23	24	36/11	52.2 ± 7.4 years old	II, III	—	—	Cinobufotalin injection + TACE (5-FU, DDP, MMC)	TACE (5-FU, DDP, MMC)	4 weeks	1. Complication 2. Comparison of liver Function and AFP
											3. Life quality
											4. Solid tumor variation
Shen J.J.^58^ 2015	18	18	23/13	E:57.5 years old	—	A and B	≥ 60	Cinobufotalin injection + TACE (Lobaplatin, DDP, MMC)	TACE (Lobaplatin, DDP, MMC)	2 weeks	1. Clinical effectiveness
				C:54.7 years old							2. Comparison of HIF-1α and VEGF
Shu X.H.^59^ 2004	29	29	48/10	Median age: 51 years	II, III	—	≥ 60	Cinobufotalin injection + 5-fluoro-uracil	5-fluorouracil	1–2 rounds (5 days per round)	1. Clinical effectiveness
											2. The degree of pain relief
											3. Life quality
											4. Complication
Su Y.^60^ 2013	33	30	53/10	E:53.2 ± 8.7 years old	II, III	—	>60	Cinobufotalin injection + TACE (5-FU, HCPT, DOX, MMC)	TACE (5-FU, HCPT, DOX, MMC)	4 weeks	1. Clinical effectiveness
				C:52.7 ± 7.9 years old							2. Life quality
											3. Complication
Sun Z.J.^61^ 2002	118	118	197/39	51.4 years old	—	—	—	Cinobufotalin injection + TACE (EPI, MMC, CBP)	TACE (EPI, MMC, CBP)	2 rounds (4 weeks per round)	1. Clinical effectiveness
											2. Survival at 12/24/36 months
											3. Immunological function
											4. Liver function and complication
Tang J.G.^62^ 1999	46	42	67/21	E:49 years old	—	—	—	Cinobufotalin injection + FAM (5-FU, DOX, MMC)	FAM (5-FU, DOX, MMC)	1 month	1. Complication
				C:48 years old							2. AFP
											3. Life quality
Tian X.L.^63^ 2006	36	36	53/19	E:53.4 ± 10.5 years old	—	—	≥ 70	Aiyishu injection + TACE (MMC, DOX, 5-FU, DDP)	TACE (MMC, DOX, 5-FU, DDP)	2–3 rounds (4–6 weeks per round)	1. Clinical effectiveness
				C:52.5 ± 9.6 years old							2. QoL scores
											3. Life quality
											4. Survival at 6/12/18/24 months
Wang C.J.^64^ 2001	30	30	—	48 years old	—	A,B and C	≥ 30	Cinobufotalin injection + TACE (DOX, CBP, MMC)	TACE (DOX, CBP, MMC)	—	1. Improvement of clinical symptom
											2. Complication
											3. Child-pugh level
											4. Serum levels of hepatic fibrosis
											5. AFP
											6. Clinical effectiveness
											7. Survival at 12/24/36 months
Wang L.J.^65^ 2007	72	70	117/25	E:62.5 years old	II, III	A and B	≥ 50	Aidi injection + FAP (5-FU, EPI, DDP)	FAP (5-FU, EPI, DDP)	2 rounds (30 days per round)	1. Clinical effectiveness
				C:64.1 years old							2. Life quality
											3. Child-pugh level
											4. AFP
Wang Q.C.^66^ 2013	24	24	41/7	E:median age 55 years	—	A and B	—	THM (toad venom, Salvia miltiorrhiza, and matrine) + TACE (MMC, THP, OXA)	TACE(MMC, THP, OXA)	3 days	Comparison of T-lymphocyte subsets
				C:median age 57.2 years							
Wei Y.F.^67^ 2015	48	44	61/31	58.6 ± 6.8 years old	—	A and B	≥ 70	Sodium cantharidinate and vitamin B6 + TACE (5-FU, EPI, MMC)	TACE (5-FU, EPI, MMC)	3 rounds (30 days per round)	1. Clinical effectiveness
											2. Complication
											3. Survival at 12/24/36 months
Wu H.M.^68^ 200	36	44	69/11	E:52.4 ± 7.2 years old	II, III	—	—	Cinobufotalin injection + TACE (5-FU, MMC, DDP)	TACE (5-FU, MMC, DDP)	2 rounds (10 days per round)	1. Clinical effectiveness
											2. AFP
				C:50.5 ± 8.7 years old							3. Comparison of tumor metastasis
											4. Survival at 3/6/12 months
											5. Complication
Wu J.Y.^69^ 2006	30	30	53/7	E:50 years old	III	—	—	Cinobufotalin injection + TACE (HCPT, THP)	TACE (HCPT, THP)	25 days	1. Clinical effectiveness
				C:50 years old							2. Complication
Wu J.S.^70^ 2015	15	15	26-Apr	44–68 years old	—	—	≥ 70	Sodium cantharidinate and vitamin B6 + TACE (5-FU, EPI, MMC)	TACE (5-FU, EPI, MMC)	3 rounds	1. Comparison of WBC, ALT and AST
											2. Complication
Wu Z.M.^71^ 2010	41	41	50/32	Median age 45 years old	—	—	—	Sodium cantharidinate and vitamin B6 + TACE (5-FU, EPI, DDP)	TACE (5-FU, EPI, DDP)	2 weeks	1. Clinical effectiveness
											2. Comparison of liver function and AFP
											3. Comparison of new vessel and portal vein tumor thrombosis
Xiao X.S.^72^ 2011	25	25	35/15	E:65.4 years old	—	—	—	Cinobufotalin injection + TACE (MMC, 5-FU, DOX, DDP, HCPT)	TACE (MMC, 5-FU, DOX, DDP, HCPT)	2 rounds (15–20 days per round)	Clinical effectiveness
				C:63.6 years old							
Xie J.^73^ 2015	50	50	89/11	E:58.09 ± 11.67 years old	—	A	—	Scorpion and earthworm + TACE (5-FU, MMC, EPI)	TACE (5-FU, MMC, EPI)	2 months	1. Numbers of TACE
				C:58.32 ± 11.55 years old							2. AFP
											3. Liver function
											4. Lung metastasis
Xie Y.F.^74^ 2003	31	31	49/13	Median age 53.5 years	I, II, III	—	>60	Jinlong capsule + TACE (5-FU, DDP, MMC)	TACE (5-FU, DDP, MMC)	21 days	1. Clinical effectiveness
											2. Life quality
											3. Comparison of WBC
											4. Immunologic function
											5. AFP
Xu Y.S.^75^ 2011	64	64	84/44	50.25 years old	—	—	>60	Sodium norcantharidate (SNCTD) + TACE (DDP, 5-FU, DOX)	TACE (DDP, 5-FU, DOX)	1–3 rounds (15 days per round)	1. Clinical effectiveness
											2. Survival at 12/24/36 months
											3. KPS
											4. Complication
Xue Q.^76^ 2010	32	30	45/17	E:45.75 ± 11.40 years old	—	—	>60	Cinobufotalin injection + TACE (DDP, 5-FU, DOX)	TACE (DDP, 5-FU, DOX)	1–3 rounds (28 days per round)	1. Clinical effectiveness
				C:45.45 ± 10.70 years old							2. Survival at 6/12/24/36 months
											3. Comparison of clinical symptoms
											4. KPS
											5. Complication
Yang P.Y.^77^ 2013	34	36	48/22	55.1 ± 8.2 years old	—	A and B	≥ 60	Jinlong capsule + TACE (EPI, 5-FU, DDP)	TACE (EPI, 5-FU, DDP)	42 weeks	1. Clinical effectiveness
											2. Comparison of ALT, TBIL and ALB
											3. KPS scores
											4. TCM clinical symptom
											5. TH1 and TH2
											6. Safety analysis
You S.Y.^78^ 2006	25	25	32/18	E:49 ± 3 years old	—	—	—	Cinobufotalin injection + TACE (5-FU, DDP, EPI)	TACE (5-FU, DDP, EPI)	2 rounds	1. Clinical effectiveness
				C:48 ± 9 years old							2. Survival at 12/24/36 months
											3. Complication
Yu J.G.^79^ 2013	30	30	29-Nov	E:49.7 years old	—	—	>60	Cinobufotalin injection + TACE (DDP, DOX, MMC, 5-FU)	TACE (DDP, DOX, MMC, 5-FU)	60 days	1. Clinical effectiveness
				C:50.8 years old							2. Clinical symptom improvement
											3. Comparison of blood routine examination, liver function and AFP
Yuan C.Y.^80^ 2007	20	20	28-Dec	E:52.3 ± 3.5 years old	II, III	—	—	Cinobufotalin injection + TACE (5-FU, DDP, EPI)	TACE (5-FU, DDP, EPI)	2 rounds	1. Clinical effectiveness
				C:53.2 ± 3.4 years old							2. AFP
											3. Life quality
											4. Immunological function
											5. Complication
Zhang B.^81^ 2007	51	49	76/24	E:54.3 years old	—	—	>80	Sodium cantharidinate and vitamin B6 + TACE (THP)	TACE (THP)	4 weeks	1. Clinical effectiveness
				C:50.1 years old							2. Life quality
											3. Clinical symptom improvement
											4. Complication
Zhang C.Q.^82^ 2005	116	108	187/37	E:52.1 ± 9.7 years old	—	A, B and C	—	Jinlong capsule + TACE (EPI, MMC, CBP)	TACE (EPI, MMC, CBP)	3 years	1. Survival at 6/12/24/36 months
				C:50.4 ± 8.5 years old							2. Clinical effectiveness
											3. QoL scores
Zhang M.J.^83^ 2011	38	38	38/38	E:53.99 ± 2.43 years old	—	—	≥ 60	Sodium cantharidinate and vitamin B6 + TACE (MMC, DOX)	TACE (MMC, DOX)	4–6 weeks	1. Clinical effectiveness
				C:55.02 ± 2.16 years old							2. Clinical symptom improvement and weight
											3. Life quality
											4. Immunological function
											5. Complication
Zhang T.S.^84^ 2011	32	32	53/11	E:52 years old	—	—	>60	Cinobufotalin injection + TACE (DOX, CBP, MMC)	TACE (DOX, CBP, MMC)	4–6 weeks	1. Clinical effectiveness
				C:51 years old							2. AFP
											3. Complication
											4. Survival at 6/12/24/36 months
Zhou J.S.^85^ 2006	21	22	34/9	60.08 years old	II, III	A, B and C	50–70	Cinobufotalin injection + TACE (5-FU, DDP, MMC)	TACE (5-FU, DDP, MMC)	4 rounds (4 weeks per round)	1. Clinical effectiveness
											2. Liver function
											3. Clinical symptom improvement
											4. Life quality
											5. Survival at 18/24 months
											6. AFP and KPS
Zhu W.Q.^86^ 2014	48	50	71/27	47.5 years old	—	—	—	Sodium cantharidinate and vitamin B6 + TACE (-)	TACE (-)	20 days	1. Complication
											2. Liver function, Child-Pugh scores
											3. Serum hepatic fibrosis markers
											4. Clinical effectiveness

Note: E: Experimental group; C: Control group; M: Male; F: Female; KPS: Karnofsky performance score; RCT: Randomized clinical trial; TACE: Transcatheter hepatic arterial chemoembolization; THM: Traditional herb medicine; AFP: Alpha-fetoprotein; Th1: Helper T cell type 1; Th2: Helper T cell type 2; HIF-1α: Hypoxia inducible factor-1α; MST: Median survival time; TTP: Time to progression; QoL: Quality of life; VEGF: Vascular endothelial growth factor; WBC: White blood cell; TBIL: Total bilirubin; ALT: Alanine aminotrnsferase; ALB: Albumin; MTTP: Median time to progression; CBR: Clinical beneficial rate; IFN: Interferon; OXA: Oxaliplatin; EPI: Epirubicin; 5-FU: 5-fluorouracil; THP: Pirarubicin; DDP: Cisplatin; CF: Calcium folinate; GEM: Gemcitabine; DOX: Doxorubicin; MMC: Mitomycin; HCPT: Hydroxycamptothecine; CBP: Carboplatin.

### Included articles characteristics and quality evaluation

A total of 57 publications met with the final eligibility criteria. All studies were randomized clinical trials (RCTs) and carried out in the hospitals in China. All trials were single-center studies except one performed in 3 different areas’ hospitals in China^45^. Published year of articles was between 1999 and 2015. These trials were conducted among men and women between 18 and 85 years old. There were eighteen articles clearly introduce the stage of HCC^30, 32, 35, 39, 40, 45, 46, 55–57, 59, 60, 65, 68, 69, 74, 80, 85^. The most common stage of HCC was the stage II-III based on the National Comprehensive Cancer Network (NCCN) guidelines 2015^21^ except for 2 publications including stage I hepatocellular carcinoma^45, 74^. (Table 1) Seventeen articles clearly reported the Child-Pugh grades included class A and B^31, 32, 37, 39, 41, 44, 45, 49–51, 53, 58, 64–67, 73^. However, Child-Pugh class C was extra included in 5 articles mentioned before^39, 44, 50, 51, 64^ and Child-Pugh class A was only included in 1 article^73^. The traditional insect Chinese medicine and the related preparation are diversified in included articles: the major insect related medicines contained as follow: cinobufotalin injection, Aidi injection, sodium cantharidinate and vitamin B6 injection, sodium norcantharidate injection, jinlong capsule, and compound cantharides capsule. (Table 1) The major chemotherapy plan in studies was transcatheter hepatic arterial chemoembolization (TACE), while some articles reported additional chemotherapy regimens, which included FAP (5-fluorouracil + doxorubicin + cisplatin), FAM (5-fluorouracil + doxorubicin + mitomycin), FOLFOX4 (oxaliplatin + calcium folinate + 5-fluorouracil) and 5-fluorouracil^30, 31, 35, 55, 59, 62, 65^. Drug types of chemotherapy for included trials were listed as follows: oxaliplatin, epirubicin, 5-fluorouracil, pirarubicin, cisplatin, calcium folinate, gemcitabine, doxorubicin, mitomycin, hydroxycamptothecine and carboplatin. (Table 1) As for the duration of therapy, all included studies except one^64^ clearly reported although the range of treating duration was various obviously. All included trials except one^86^ clearly introduce the dosage of chemotherapy drugs.

**Figure 2:**
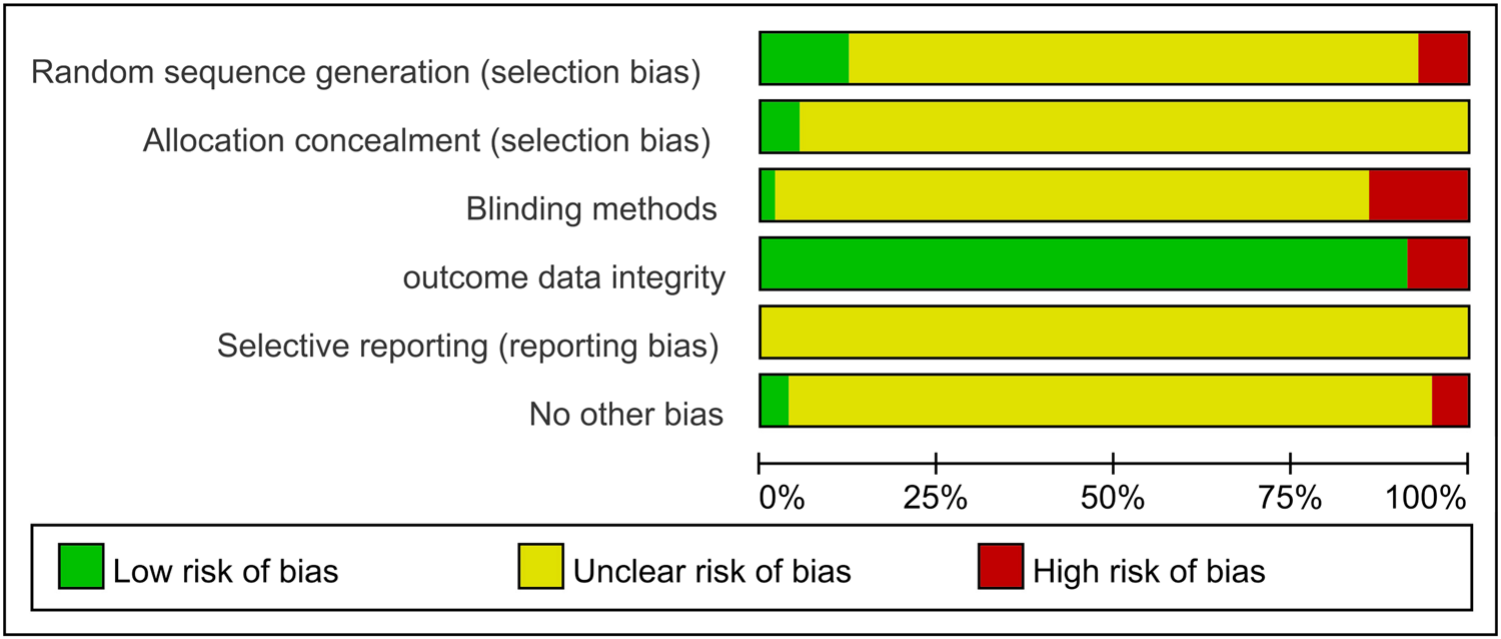
The risk of bias graph.

**Figure 3:**
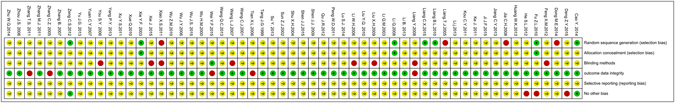
The risk of bias summary.

For the included articles, all of them expect 7 studies (12%) were only mentioned the allocation sequence generation without showing the specific random method. However, of the 7 articles, three used shuffling envelopes method^36, 45, 74^ and 4 used the random number table method^31, 32, 48, 49^. Only 3 articles (5%) mentioned the allocation concealment from the included studies: the sealed envelope method was the way they chosen^36, 45, 74^. All articles did not report the blinding method, excepted 1 article which was a single-blind randomized clinical trial^70^. Most of studies (92%) were ranked as low risk of outcome integrity data, while 5 articles (8%) reported participants’ dropout reason^51, 63, 67, 82, 84^. None of the articles clearly illustrated the reporting bias and only 2 studies (3%) were rated as low risk for no other bias^31, 32^. (Table 2, Figs 2, 3).

**Table 2:** The Risk Bias Evaluation of Articles

Article names	Random collection method	Allocation concealment	The blinding method	Outcome data integrity	The outcome data of selective report	no other bias sources	The level of bias risk
Huang W.K.^30^ 2013	U	U	U	Y	U	U	High
Cao Y.^31^ 2014	Y	U	U	Y	U	Y	Low
Zeng C.S.^32^ 2012	Y	U	U	Y	U	Y	Low
Dong M.E.^34^ 2014	N	U	U	Y	U	U	High
Feng X.M.^35^ 2012	U	U	N	Y	U	U	High
Fu Z.L.^36^ 2010	Y	Y	U	Y	U	N	Low
He S.L.^37^ 2012	U	U	U	Y	U	N	High
Ji J.F.^38^ 2015	U	U	U	Y	U	U	High
Jia C.H.^39^ 2008	N	U	U	Y	U	U	High
Jiang C.Y.^40^ 2013	U	U	U	Y	U	U	High
Ke J.^41^ 2011	U	U	U	Y	U	U	High
Kou C.Y.^42^ 2011	U	U	U	Y	U	U	High
Li B.^43^ 2013	U	U	U	Y	U	U	High
Li J.^44^ 2013	U	U	U	Y	U	U	High
Li Q.^45^ 2008	Y	Y	U	Y	U	U	Low
Li Q.M.^46^ 2003	U	U	U	Y	U	U	High
Li W.H.^47^ 2006	U	U	N	Y	U	U	High
Liang B.L.^48^ 2010	Y	U	U	Y	U	U	High
Liang C.X.^49^ 2015	Y	U	U	Y	U	U	High
Liang T.J.^50^ 2005	N	U	U	Y	U	U	High
Liang Y.^51^ 2008	U	U	N	N	U	U	High
Liu X.H.^52^ 2009	U	U	N	Y	U	U	High
Liu Y.Q.^53^ 2010	U	U	U	Y	U	U	High
Lu S.J.^54^ 2014	U	U	U	Y	U	U	High
Peng W.D.^55^ 2011	U	U	U	Y	U	U	High
Qu J.R.^56^ 2012	U	U	U	Y	U	U	High
Shen J.J.^57^ 2009	U	U	U	Y	U	U	High
Shen J.J.^58^ 2015	U	U	U	Y	U	U	High
Shu X.H.^59^ 2004	U	U	U	Y	U	U	High
Su Y.^60^ 2013	U	U	U	Y	U	U	High
Sun Z.J.^61^ 2002	U	U	U	Y	U	U	High
Tang J.G.^62^ 1999	U	U	U	Y	U	U	High
Tian X.L.^63^ 2006	U	U	U	N	U	U	High
Wang C.J.^64^ 2001	U	U	U	Y	U	U	High
Wang L.J.^65^ 2007	U	U	N	Y	U	U	High
Wang Q.C.^66^ 2013	U	U	U	Y	U	U	High
Wei Y.F.^67^ 2015	U	U	Y	N	U	U	High
Wu H.M.^68^ 2000	U	U	U	Y	U	U	High
Wu J.Y.^69^ 2006	U	U	U	Y	U	U	High
Wu J.S.^70^ 2015	U	U	U	Y	U	U	High
Wu Z.M.^71^ 2010	U	U	U	Y	U	U	High
Xiao X.S.^72^ 2011	N	U	N	Y	U	U	High
Xie J.^73^ 2015	U	U	N	Y	U	U	High
Xie Y.F.^74^ 2003	Y	Y	U	Y	U	U	Low
Xu Y.S.^75^ 2011	U	U	U	Y	U	U	High
Xue Q.^76^ 2010	U	U	U	Y	U	U	High
Yang P.Y.^77^ 2013	U	U	U	Y	U	U	High
You S.Y.^78^ 2006	U	U	N	Y	U	U	High
Yu J.G.^79^ 2013	U	U	U	Y	U	U	High
Yuan C.Y.^80^ 2007	U	U	U	Y	U	U	High
Zhang B.^81^ 2007	U	U	U	Y	U	U	High
Zhang C.Q.^82^ 2005	U	U	U	N	U	U	High
Zhang M.J.^83^ 2011	U	U	U	Y	U	U	High
Zhang T.S.^84^ 2011	U	U	U	N	U	U	High
Zhu W.Q.^85^ 2014	U	U	U	Y	U	U	High
Deng Z.Y.^33^ 2015	U	U	U	Y	U	N	High
Zhou J.S.^86^ 2006	U	U	U	Y	U	U	High

Note: Y refers to “yeas”; N refers to “no”; U refers to “unclear”.

### Meta-analysis

#### Objective response rates (CR + PR)

**Figure 4:**
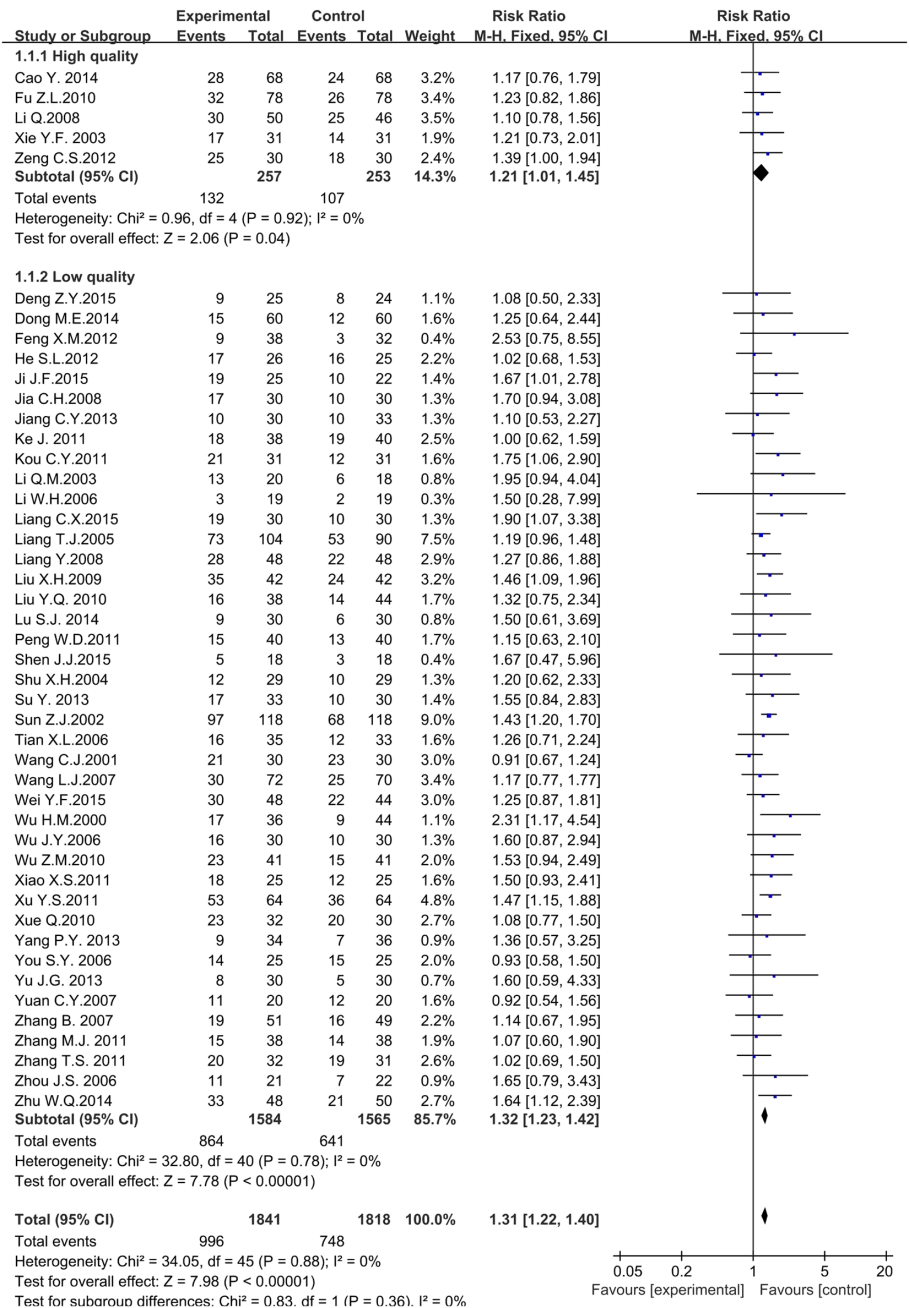
The forest plot of subgroup analysis based on the article’s quality for the objective response rate of traditional insect Chinese medicine and the related preparation combined chemotherapy versus chemotherapy alone (*M-H*: Mantel-Haenszel estimates; *CI*: Confidence Interval).

Figure 4 illustrated the clinical effects (objective response rate) of traditional insect Chinese medicine in advanced hepatocellular carcinoma (HCC) therapy. There were 46 articles including 3,659 participants analyzed in the forest plot^31–42, 45–47, 49–55, 57–61, 63–65, 67–72, 74–81, 83–86^. We conducted subgroup analysis on the condition of the pervasive low quality (high bias risk) of included studies. We extracted the high quality articles (5 papers, 510 participants) from the 46 papers based on the Cochrane Collaboration’s tools for assessing risk of bias mentioned before ^31, 32, 36, 45, 74^, making meta-analysis to compare with the rest low quality articles (41 papers, 3,149 participants). The meta-analysis reported that the objective response rate (CR + PR) of high quality group, low quality group and overall group all revealed traditional insect Chinese medicine combined chemotherapy was superior to single chemotherapy method for the non-surgical HCC (Low quality: *RR* = 1.21, 95% *CI* = 1.01 to 1.45, *P* = 0.04 < 0.05; High quality: *RR* = 1.32, 95% *CI* = 1.23 to 1.42, *P* < 0.001; Overall: *RR* = 1.31, 95% *CI* = 1.22 to 1.40, *P* < 0.001). The results showed that the objective response rate for the treatment group and the control group has statistical difference. The heterogeneity did not exist in the trials (Low quality: *P* = 0.92, *I* ^*2*^ = 0%; High quality: *P* = 0.78, *I* ^*2*^ = 0%; Overall: *P* = 0.88, *I* ^*2*^ = 0%) and the fixed effects model was performed to calculate combined data by Mantel-Haenszel test.

#### Survival time

**Figure 5:**
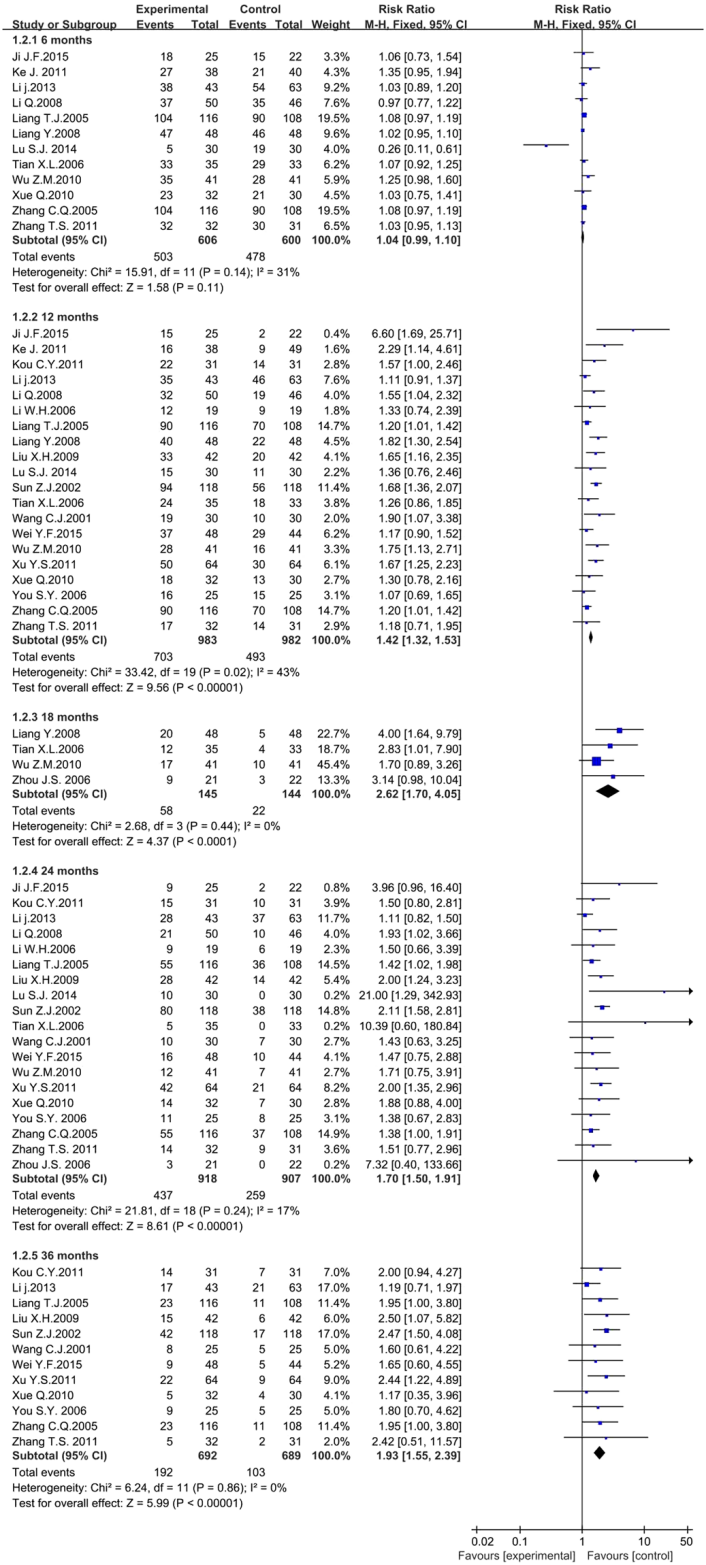
The forest plot of survival time for traditional insect Chinese medicine and the related preparation combined chemotherapy versus chemotherapy alone (*M-H*: Mantel-Haenszel estimates; *CI*: Confidence Interval).

Five kinds of survival time were assessed in the meta-analysis including 6 months, 12 months, 18 months, 24 months and 36 months. (Fig. 5). Twelve articles including 1,206 participants analyzed the 6 months survival time^38, 41, 44, 45, 50, 51, 54, 63, 71, 76, 82, 84^. The meta-analysis showed the 6 months survival time for the experimental group (used traditional insect Chinese medicine combined chemotherapy) and the control group (used chemotherapy alone) was irrelevant (*RR* = 1.04, 95% *CI* = 0.99 to 1.10, *P* = 0.14å 0.05). The heterogeneity was moderately high (*P* = 0.14, *I* ^*2*^ = 31%), which might be due to a particular trial^54^ included in the meta-analysis. So we performed the sensitivity analysis and if this trial was excluded, the *I* ^*2*^ value would be down to 0%. The fixed effects model was performed to pool data. Twenty articles including 1,831 participants assessed the 12 months survival time^38, 41, 42, 44, 45, 47, 50–52, 54, 61, 63, 64, 67, 71, 75, 76, 78, 82, 84^. The meta-analysis illustrated that the experimental group improved the 12 months survival time compared with the control group (*RR* = 1.42, 95% *CI* = 1.32 to 1.53, *P* < 0.001). The heterogeneity was moderately high (*P* = 0.02, *I* ^*2*^ = 43%) while the *I* ^*2*^ value was lower than 50%. The fixed effects model was performed to pool data. Only four articles including 289 participants analyzed the 18 months survival time^51, 63, 71, 85^. The meta-analysis illustrated the experimental group significantly improved the 18 months survival time compared with the control group (*RR* = 2.62, 95% *CI* = 1.70 to 4.05, *P* < 0.001). There was no heterogeneity in these four articles (*P* = 0.44, *I* ^*2*^ = 0%), so the fixed effects model was performed to pool data. Nineteen articles including 1,825 participants analyzed the 24 months survival time^38, 42, 44, 45, 47, 50, 52, 54, 61, 63, 64, 67, 71, 75, 76, 78, 82, 84, 85^. The meta-analysis illustrated the experimental group significantly improved the 24 months survival time compared with the control group (*RR* = 1.70, 95% *CI* = 1.50 to 1.91, *P* < 0.001). The heterogeneity was low among the included articles (*P* = 0.24, *I* ^*2*^ = 17%) and the fixed effects model was performed to pool data. Twelve articles including 1,381 participants analyzed the 36 months survival time^42, 44, 52, 61, 64, 67, 75, 76, 78, 82, 84^. The meta-analysis illustrated the experimental group significantly improved the 36 months survival time compared with the control group (*RR* = 1.93, 95% *CI* = 1.55 to 2.39, *P* < 0.001). There was no heterogeneity in these 4 articles (*P* = 0.86, *I* ^*2*^ = 0%) and the fixed effects model was performed to pool data. We did not find any valid articles that had analyzed the 5 years survival time, and only one article analyzed 3 months survival time^68^.

#### Indexes improvement for QoL scores, KPS, and AFP

**Figure 6:**
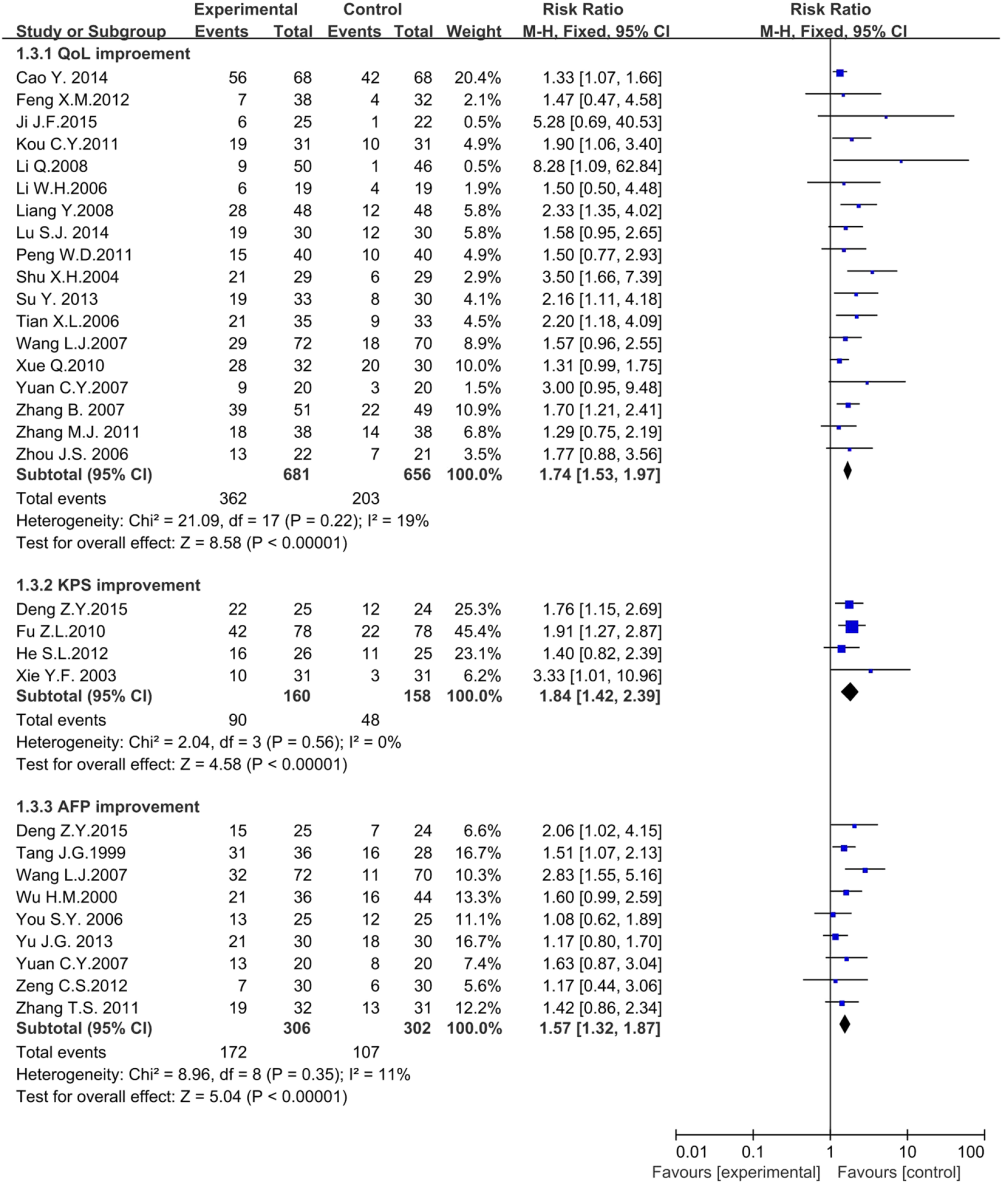
The forest plot of QoL scores, KPS and AFP improvement for traditional insect Chinese medicine and the related preparation combined chemotherapy versus chemotherapy alone (QoL: Quality of life; KPS: Karnofsky performance scores; AFP: Alpha fetoprotein; *M-H*: Mantel-Haenszel estimates; *CI*: Confidence Interval).

Eighteen trials including 1,337 patients reported data on quality of life (QoL) scores improvement^31, 35, 38, 42, 45, 47, 51, 54, 55, 59, 60, 63, 65, 76, 80, 81, 83, 85^. No obvious heterogeneity was found in the included articles (*P* = 0.22, *I* ^*2*^ = 19%), so that the fixed effects model was conducted to pool data. The meta-analysis showed that the QoL scores improvement in the experimental group was superior to the control group (*RR* = 1.74, 95% *CI* = 1.53 to 1.97, *P* < 0.001). Only 4 trials including 318 patients reported data on Karnofsky performance scores (KPS) improvement^33, 36, 37, 74^, and 9 trials including 608 patients on AFP improvement^32, 33, 62, 65, 68, 78–80, 84^. Both heterogeneities of the two indexes were not notable (KPS: *P* = 0.56, *I* ^*2*^ = 0%; AFP: *P* = 0.35, *I* ^*2*^ = 11%) and the statistical model was fixed effects model. The meta-analysis showed both the two indexes (KPS and AFP) were significantly improved in experimental group compared with the control group (KPS: *RR* = 1.84, 95% *CI* = 1.42 to 2.39, *P* < 0.001; AFP: *RR* = 1.57, 95% *CI* = 1.32 to 1.87, *P* < 0.001). (Fig. 6).

#### Side effects

**Figure 7:**
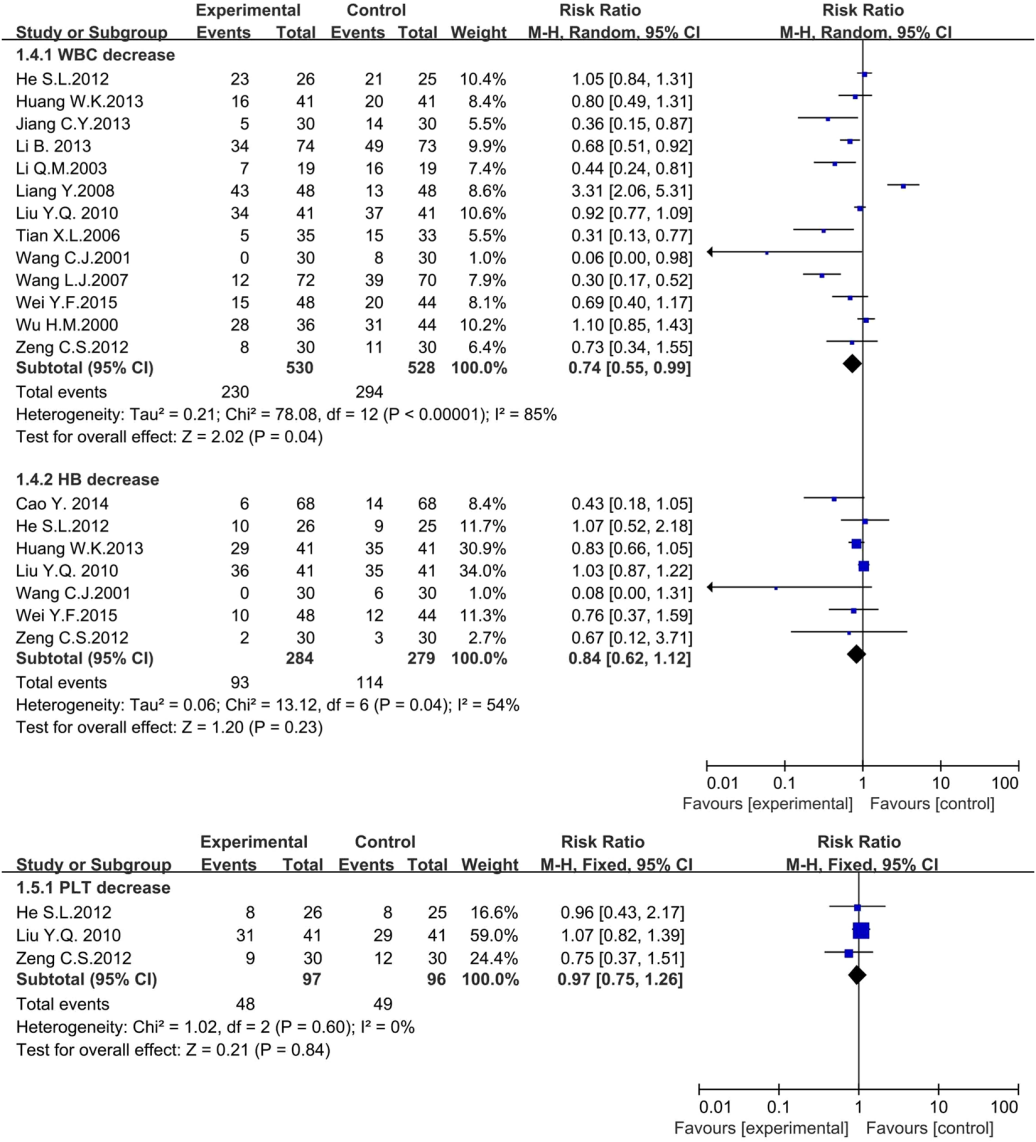
The forest plots of bone marrow depression (WBC decrease, HB decrease and PLT decrease) for traditional insect Chinese medicine and the related preparation combined chemotherapy versus chemotherapy alone (PLT: Platelets, WBC: White blood cell, HB: Hemoglobin, *M-H*: Mantel-Haenszel estimates; *CI*: Confidence Interval).

This part of meta-analysis included 6 kinds of side effects, which contained bone marrow depression, gastrointestinal adverse reactions, liver damage, kidney damage, fever and pain. As for bone marrow depression, two laboratory indexes including white blood cell (WBC) decrease (13 articles, 1058 patients)^30, 32, 37, 40, 43, 46, 51, 53, 63–65, 67, 68^ and hemoglobin (HB) decrease (7 articles, 563 patients)^30–32, 37, 53, 64, 67^ were applied as random effects model (the heterogeneity of WBC decrease: *P* < 0.001, *I* ^*2*^ = 85%; HB decrease: *P* = 0.04, *I* ^*2*^ = 54%); one laboratory index named the platelets (PLT) decrease (only 3 articles, 193 patients)^32, 37, 53^ was applied fixed effects model (heterogeneity: *P* = 0.6, *I* ^*2*^ = 0%). The subgroup analysis (seen in the Supplementary Information of the article) of WBC decrease was performed based on the drugs classification, literature publication years and experimental areas, but the source of heterogeneity was still unclear. However, the subgroup analysis of HB decrease found that the heterogeneity came from experimental areas (North of China: *P* = 0.27, *I* ^*2*^ = 23%; South of China: *P* = 0.13, *I* ^*2*^ = 46%). The incidence rates of HB decrease and PLT decrease in experimental group were irrelevant to control group with corresponding Risk Ratio (*RR*) and 95% *CI* were (0.84, 0.62to 1.12, *P* = 0.23å 0.05), and (0.97, 0.75 to 1.26, *P* = 0.84å 0.05), respectively. The incidence rate of WBC decrease in the experiment group was lower than in control: (0.74, 0.55 to 0.99, *P* = 0.04 < 0.05). (Fig. 7). Red blood cell (RBC) decrease, another important component of bone marrow depression, was excluded owing to only one trial analyzing it^30^.

Twenty articles including 1,529 patients reported data on gastrointestinal adverse reactions^31–34, 40, 46, 53, 58, 59, 64, 65, 67, 69, 75, 78–81, 83, 86^. The random effects model was conducted to pool data because the heterogeneity (*P* < 0.001, *I* ^*2*^ = 62%) was obvious. We made the subgroup analysis based on the drugs categories, publication years and experimental areas. The outcome of subgroup analysis showed that the variation among the different experimental areas was the source of heterogeneity (North of China: *P* = 0.34, *I* ^*2*^ = 11%; South of China: *P* = 0.18, *I* ^*2*^ = 28%). The meta-analysis showed the gastrointestinal adverse reactions in the experimental group were significantly fewer than the control group (*RR* = 0.63, 95% *CI* = 0.51 to 0.77, *P* < 0.001).

Eleven articles including 815 patients and 5 articles including 340 participants gathered information on liver damage^33, 34, 40, 51, 53, 67, 73, 78, 80, 81, 83^ and kidney damage^53, 67, 78, 80, 83^ respectively. The random effects model was performed to collect data for the liver damage group because the heterogeneity was high (*P* < 0.001, *I* ^*2*^ = 92%). The subgroup analysis based on the drugs categories, publication years and experimental areas didn’t reveal the source of heterogeneity. The fixed effects model was conducted to collect data for kidney damage group because no obvious heterogeneity was found in the included articles (*P* = 0.97, *I* ^*2*^ = 0%). The meta-analysis demonstrated that the liver damage and kidney damage in the experimental group were irrelevant to control group (liver damage: *RR* = 0.56, 95% *CI* = 0.31 to 1.02, *P* = 0.06 > 0.05; kidney damage: *RR* = 0.55, 95% *CI* = 0.24 to 1.27, *P* = 0.16 > 0.05).

**Figure 8:**
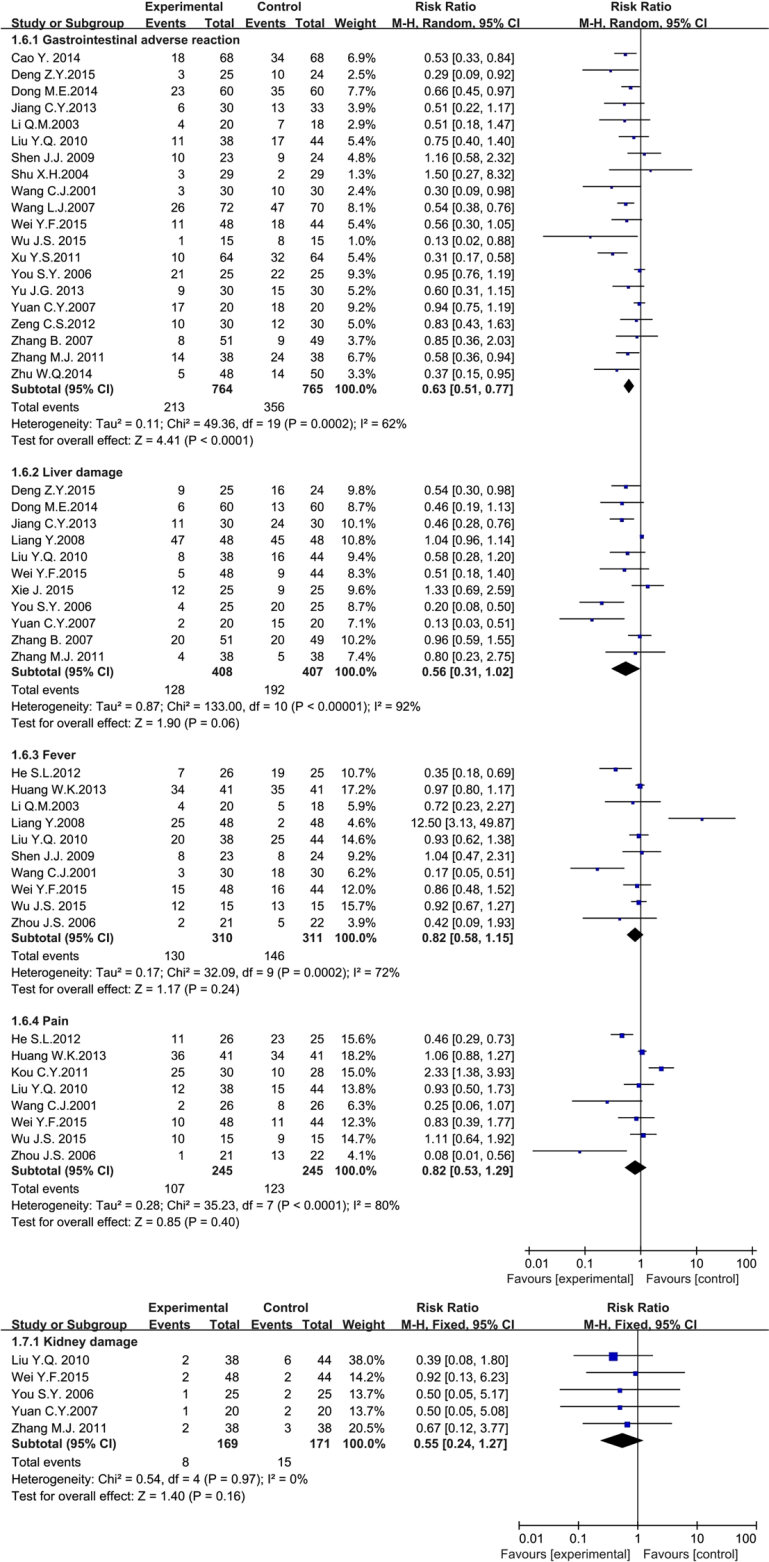
The forest plots of other side effects (gastrointestinal adverse reaction, liver damage, fever, pain and kidney damage) for traditional insect Chinese medicine and the related preparation combined chemotherapy versus chemotherapy alone (*M-H*: Mantel-Haenszel estimates; *CI*: Confidence Interval).

Ten articles including 621 patients^30, 37, 46, 51, 53, 57, 67, 69, 70, 85^ and eight articles including 490 patients^30, 37, 42, 53, 64, 67, 70, 87^ reported fever and pain respectively. They all used the random effects model for the reason of the obvious heterogeneity (*P* < 0.001, *I* ^*2*^ = 72%; *P* < 0.001, *I* ^*2*^ = 80%). We also didn’t find the source of heterogeneity through the subgroup analysis. The meta-analysis also showed both these two indexes in the treatment group and the control group were irrelevant (*RR* = 0.82, 95% *CI* = 0.58 to 1.15, *P* = 0.24 > 0.05; *RR* = 0.82, 95% *CI* = 0.53 to 1.29, *P* = 0.40 > 0.05). (Fig. 8).

#### Immune function

**Figure 9:**
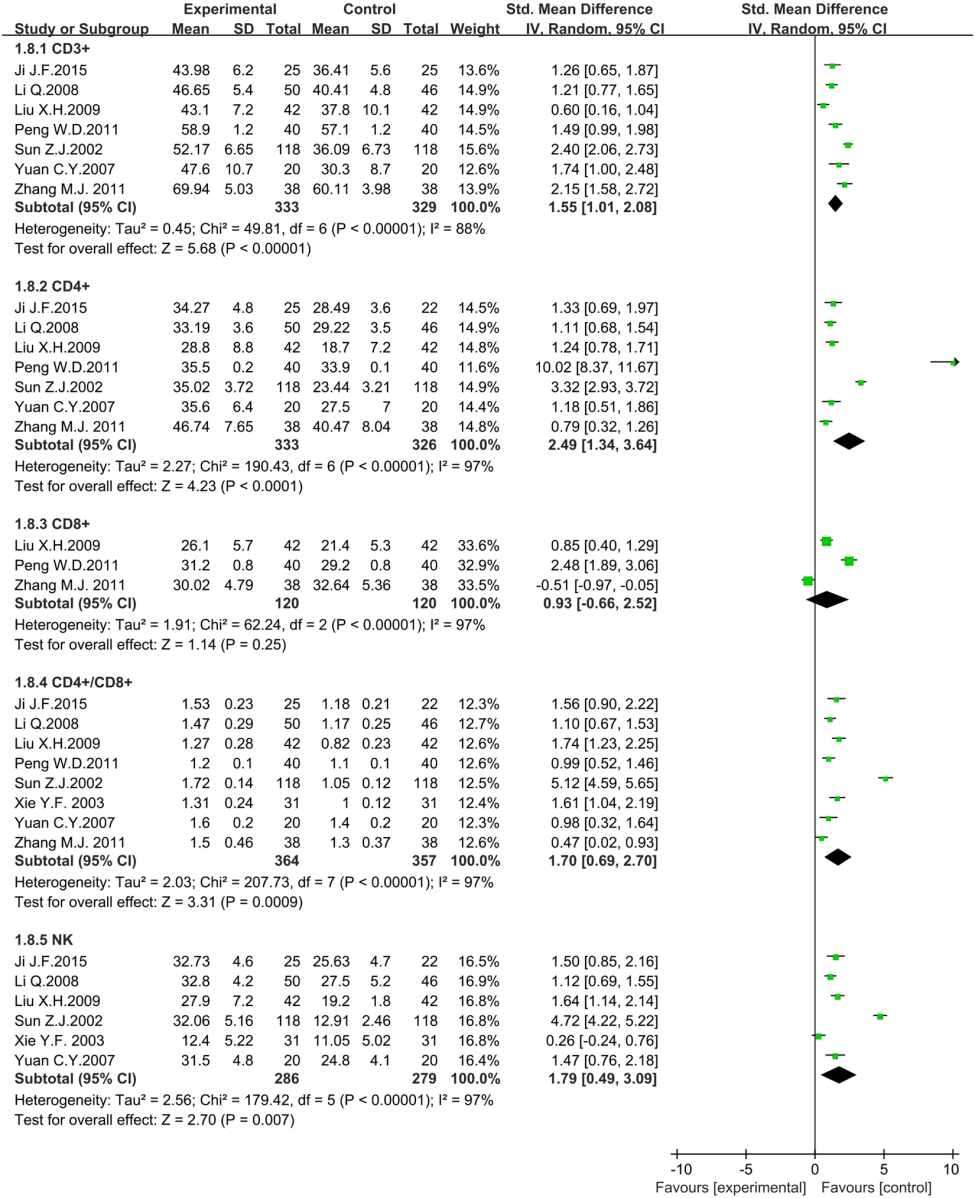
The forest plots of immune function (CD3+, CD4+, CD8+, CD4+/CD8+, NK) for traditional insect Chinese medicine and the related preparation combined chemotherapy versus chemotherapy (*M-H*: Mantel-Haenszel estimates; *CI*: Confidence Interval; NK: Natural Killer cells).

Immune function after tumor therapy was measured by continuous laboratory data as follows: CD3^+^, CD4^+^, CD8^+^, CD4^+^/CD8^+^ and Natural Killer cell (NK). As the difference of standard deviation (SD) in the same article laboratory indexes was obvious, Standard Mean Difference (Std.MD) was conducted as combined statistics. Significant improvement of CD3^+^ (7 studies, 662 patients)^38, 45, 52, 55, 61, 80, 83^, CD4^+^ (7 studies, 659 patients)^38, 45, 52, 55, 61, 80, 83^, CD4^+^/CD8^+^ (8 studies, 721 patients)^38, 45, 52, 55, 61, 74, 80, 83^ and NK (6 studies, 565 patients)^38, 45, 52, 61, 74, 80^ in the experimental group were found in the forest plot compared with the control group with associated Std.MD (95% *CI*) were 1.55 (1.01 to 2.08, *P* < 0.001), 2.49 (1.34 to 3.64, *P* < 0.001), 1.70 (0.69 2.70, *P* < 0.001) and 1.79 (0.49 to 3.09, *P* = 0.007). But CD8^+^ (3 studies, 240 patients)^52, 55, 83^ in the experimental group was irrelevant to control group with associated Std.MD (95% *CI*) was 0.93 (−0.66 to 2.52, *P* = 0.25 > 0.05). All heterogeneity that was high (*P* < 0.001, *I* ^*2*^ = 88%; *P* < 0.001, *I* ^*2*^ = 97%; *P* < 0.001, *I* ^*2*^ = 97%; *P* < 0.001, *I* ^*2*^ = 97%; *P* < 0.001, *I* ^*2*^ = 97%) might be attributed to the differences in the measurement time, measurement areas or the ingredient of traditional insect Chinese medicine. The random effects models were conducted to pool data and so any conclusions need to be made with caution (Fig. 9).

#### Network contribution graphs of included studies

**Figure 10:**
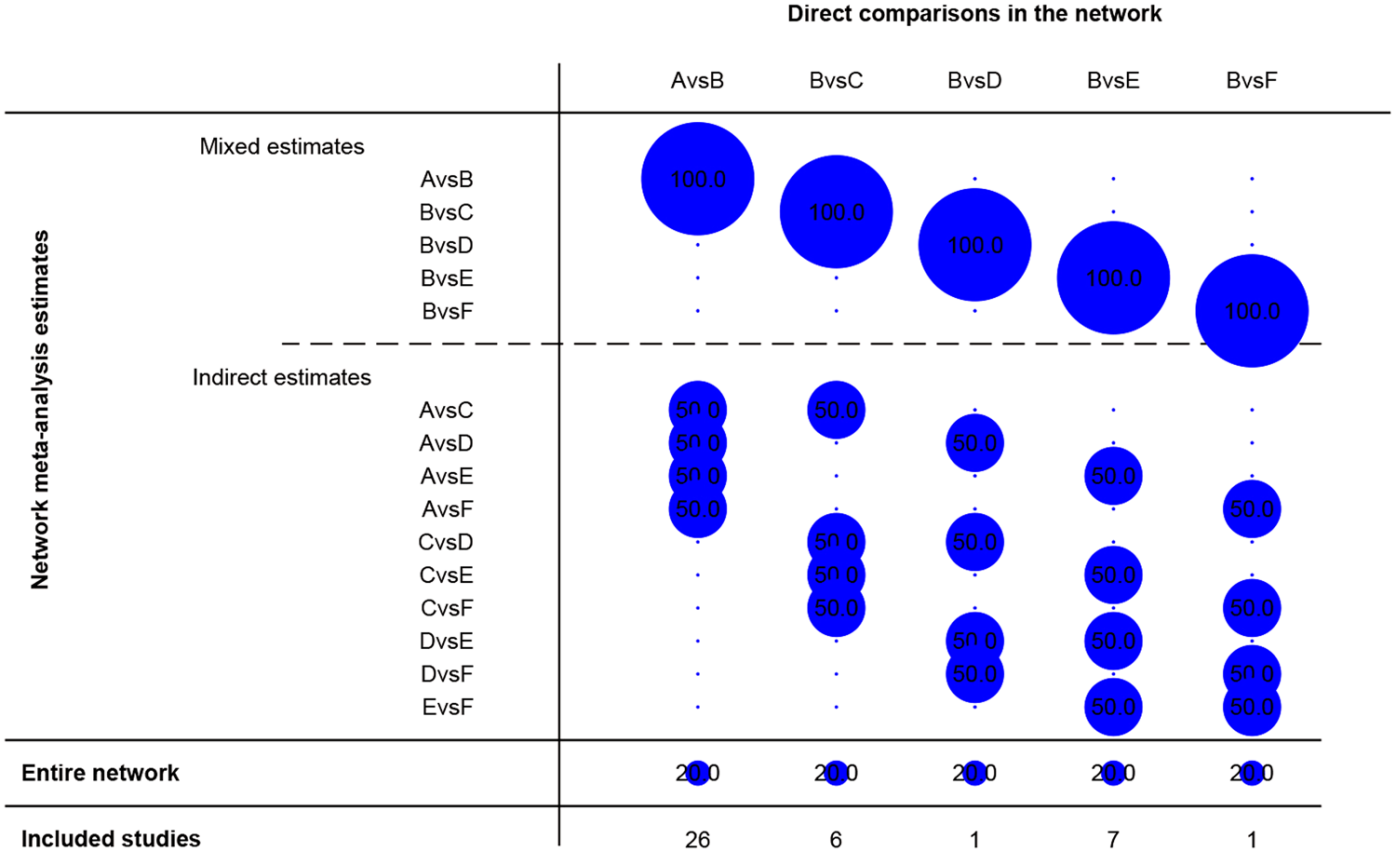
Network Contribution Graph: as for A vs B, for example, the contribute proportion of “A vs B” for “A vs B” is 100%, and for “A vs C”, “A vs D”, “A vs E” and “A vs F” was 50% respectively. The contribute proportion for entire network was 20% and 26 clinical studies included. Note: (**A**) cinobufotalin injection + TACE; (**B**) TACE; (**C**) Jinlong capsule + TACE; (**D**) Aidi injection + TACE; (**E**) Sodium Cantharidinate and Vitamin B6 Injection + TACE; F: Sodium Demethylcantharidate (SNCTD) + TACE.

Figure 10 was the network contribution graph of the experimental group and the control group. This figure illustrated the treating method numbers in orders: cinobufotalin injection plus TACE versus TACE contained 26 studies, sodium cantharidinate vitamin B_6_ injection plus TACE versus TACE contained 7 studies, Jinlong capsule plus TACE versus TACE contained 6 studies, and both Aidi injection plus TACE and sodium demethylcantharidate (SNCTD) plus TACE versus TACE contained 1 study, respectively. Five treating methods contributed 20% respectively to the entire network graph.

#### Funnel plot characteristics

**Figure 11:**
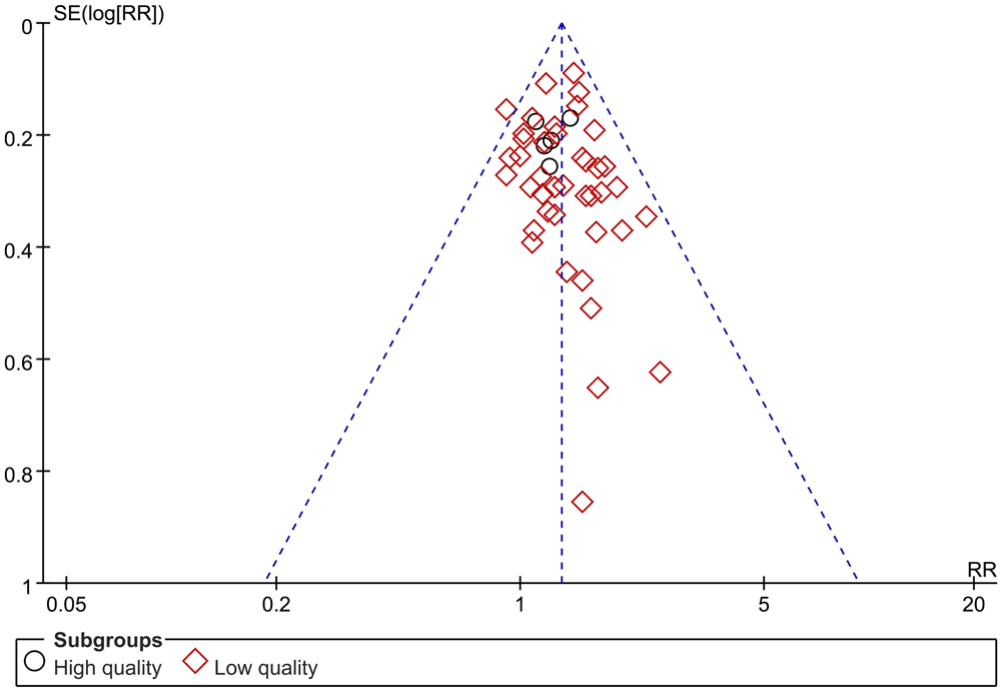
The funnel plot of objective response rate (CR+PR) Note: CR: Complete response rate. PR: Partial response rate.

**Figure 12:**
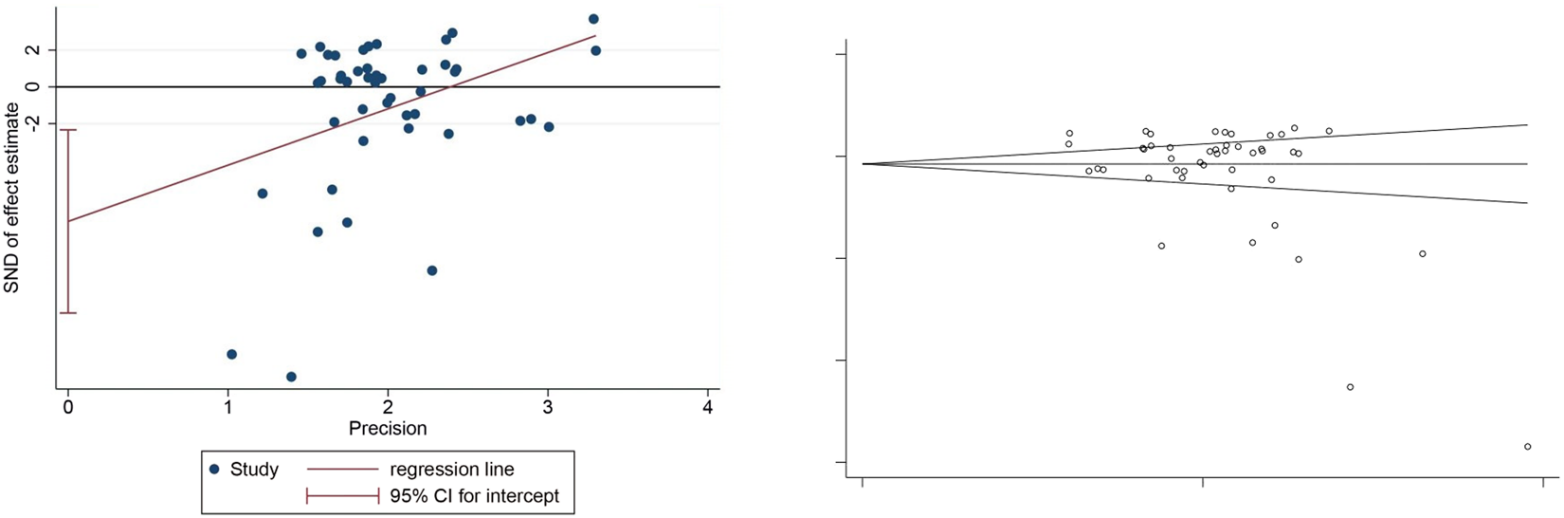
The Egger’s and Begg’s test of objective response rate (CR+PR).

We applied the pooled odds ratio (OR) as the midpoint to draw the funnel plot (Fig. 11). The publication bias was evaluated in the funnel plot by comparing the symmetry of included studies on objective response rate. The funnel plot was symmetrical in visual while the images (Fig. 12) and calculating results of Egger’s test (*t* = −2.96, *P* = 0.005) and Begg’s test (*z* = 1.96, *P* = 0.092) indicated the potential publication bias did exist.

## Discussion

Hepatocellular carcinoma (HCC) is a worldwide serious disease, causing serious health problems to human beings. The treating methods of western medicine, which include liver transplantation, liver resection, radiofrequency ablation (RFA), transcatheter hepatic arterial chemoembolization (TACE) and molecular targeted drugs, have already been used on HCC therapy for a long time. As for non-surgical hepatocellular carcinoma, the effective clinical evidence is insufficient and the heated debate also was aroused for the selection of optimal treating methods^88, 89^. The current chemotherapy performed to end-stage HCC has been proved many side effects: bone marrow depression, gastrointestinal adverse reactions, liver damage, kidney damage, fever, pain, hair loss, dizziness, *et al*. These complications also become a huge burden for HCC patients. Based on this serious context, various complementary and alternative medicine (CAM) treatments have been administered for HCC in clinical practice. The traditional insect Chinese medicine, as an essential part of TCM, has been utilized to treat HCC as an auxiliary method (or CAM methods) for a long time. The effective and safe evaluation of traditional insect Chinese medicine for non-surgical HCC treatment is urgent and necessary. To the best of our knowledge, this article is the first PRISMA compliant systemic reviews and meta-analysis to evaluate the effects and safety of traditional insect Chinese medicine combined chemotherapy for non-surgical HCC. Fifty-seven articles, all of which were conducted in China, included 4,651 participants in the meta-analysis. Even with strong cultural and population bias, seven obvious advantages still need to be emphasized: (a) fourteen medical databases were utilized and the publication language was English, Chinese and Japanese; (b) the article quality was strictly accessed and evaluated by three cooperated reviewers (T.B.Song, Y.Wan and L. Shang); (c) we performed a comprehensive analysis for different categories of the including traditional insect Chinese medicine and the subgroup analysis and sensitivity analysis was both well conducted to seek the source of heterogeneity; (d) we contacted authors on whether the included articles could receive on full-text; (e) Egger’s test, Begg’s test and funnel plot was performed to confirm whether the publication bias existed in the studies; (f) the contribution network plot graph was strictly designed to evaluate the mutual effectiveness among the included traditional insect Chinese medicines as clearly exhibited as graphs; (g) the Cochrane risk of bias tool was performed to assess the study quality. The studies’ outcomes showed good qualities were still insufficient for the reason of the high risk on blinding methods bias, selective reporting bias, and other bias.

The meta-analysis was carried out in 46 articles to evaluate the objective response rate (CR + PR), showing the significantly effectiveness in the experimental group (traditional insect Chinese medicine combined chemotherapy) compared with the control group (chemotherapy) alone. The subgroup analysis based on the articles’ quality difference also provided the same result. These results suggest that the short-term effects of traditional insect Chinese medicine as auxiliary method combined chemotherapy are superior to chemotherapy alone. It is important to note that the publication bias on objective response rate indicates inconformity between funnel plot in visual appearance and Egger’s/Begg’s test results. Involved studies showed that experimental groups significantly improve the survival time at 12 months, 18 months, 24 months and 36 months compared with control groups, revealing that traditional insect Chinese medicine combined chemotherapy might prolong the survival time of non-surgical HCC patients. However, no statistical difference was found in 6 months survival time. The meta-analysis contained quality of life (QoL) scores, Karnofsky performance scores (KPS) and AFP improvement, all showing significant improvement in the experimental group compared with the control group. We did not observe large heterogeneity among these studies and the results indicated an excellent improvement of patients’ life quality for the the utilization ﻿oftraditional insect Chinese medicine. The meta-analysis evaluated the side effects ratio after therapy, clearly showing the obvious reduction of gastrointestinal adverse reaction and WBC decrease in the experimental group compared to the control group. The liver damage, kidney damage, fever, pain, HB decrease and PLT decrease did not show significantly statistically difference although all of them indicated the same conclusion that side effects ratio of traditional insect Chinese medicine combined chemotherapy lower than chemotherapy alone. Although we found that the obvious heterogeneity in WBC decrease, HB decrease, gastrointestinal adverse reactions, liver damage, fever and pain was obvious, most of them did not show the source of heterogeneity except the HB decrease and gastrointestinal adverse reactions. The heterogeneity of HB decrease and gastrointestinal adverse reactions both came from the variation among the different experimental areas. The meta-analysis showed that the improvement of immune function in the experimental group was higher than the control group although the heterogeneity was obvious. We did not find the source of heterogeneity of them. So we could not give the firmly conclusion on the effectiveness of immune function improvement except we could include more relevant articles in the future meta-analysis. For forty-six included trials focused on objective response ratio, all experimental groups utilized traditional insect Chinese medicine related preparation as auxiliary therapy combined chemotherapy for the advanced HCC therapy.

The included traditional insect Chinese medical preparations contained cinobufotalin injection (28 trials, 62%), Jinlong capsule (6 trials, 13%), sodium cantharidinate and vitamin B6 injection (7 trials, 15%), Aidi injection (1 trial, 2%), sodium demethylcantharidate (SNCTD) (1 trial, 2%), compound cantharis capsule (1 trial, 2%), Aiyishu injection (1 trial, 2%) and Qining injection (1 trial, 2%). It has been clearly revealed that the cinobufotalin injection contributes as the major adjuvant therapy choice for non-surgical HCC. Transcatheter hepatic arterial chemoembolization (TACE) was ranked as the most frequently way of chemotherapy both in experimental groups and control groups (43 trials, 93%). Chemotherapy drugs, applied in HCC patients’ therapy through the way of TACE with 1-4 of them, were diversified, such as OXA (oxaliplatin), DDP (cisplatin), CBP (carboplatin), lobaplatin, EPI (epirubicin), DOX (doxorubicin), THP (pirarubicin), MMC (mitomycin) and HCPT (hydroxycamptothecine). The dosage of traditional insect Chinese medicine and chemotherapy drugs had their own differences.

The publication bias for objective response rate did exist in this meta-analysis after Egger’s and Begg’s test. It might be derived from (a) some authors tended to deliver positive results of articles to editors while prejudiced negative results^90^; (b) some magazine editors or reviewers had a preference to positive results of articles while caviled to negative results to some extent^87^; (c) trials that received government funding had more possibility to publish on some magazines than received private or company funding^91^. The meta-analysis would overstate the degree of association between treating effects and risk factors because of the publication bias, which would bring mistakes for clinical patient’s therapy or health decision-making.

### Limitation

There are several potential limitations of current data and results that should be interpreted with caution. Firstly, included clinical studies all conducted in different centers in China, which may bring the regional and cultural bias and influence the widely application of traditional insect Chinese medicine in the future. Secondly, for the reason of varied medical development in different regions of China, clinical centers in China conducting the trials have different clinical levels of tumor diagnosis and treatment, which could result in a bias of the reported incidence rate. Thirdly, the included randomized clinical trials have many flaws caused by human baseline risk factors (all patients were Chinese), incomplete methodological design of trials, small sample trials (some studies have less than 30 patients per group) and index criteria deficiency (the information of the clinical stage of HCC or Child-Pugh scores deficiency). Fourthly, some results showed significantly heterogeneity among the included studies, which may be due to the sample size, different experimental region in China, medicine application and dose, publication years, as well as duration of treatment. However, we need to understand that establishing the random-effects model to pool data cannot give exact and stable conclusion in this situation. Fifthly, the application of traditional insect Chinese medical preparations varied in included articles. For example, some insect medical preparations were used through the way of injection (i.e. cinobufotalin injection, sodium cantharidinate and vitamin B6 injection, Aidi injection, *et al*.) and some were administrated through the oral way (i.e. Jinlong capsule, compound cantharis capsule, *et al*.). The time of applying medicines was unsynchronized, which also might exert potential time bias.

## Conclusion

This system review and meta-analysis offers a practical and beneficial result for the effectiveness and safety of traditional insect Chinese medicine combined chemotherapy on non-surgical hepatocellular carcinoma (HCC). This combined therapy can provide assistance for improving the objective response rate (CR + PR) of HCC, prolonging long term survival time (12, 18, 24, 36 months), enhancing life quality (QoL scores and KPS) of patients, increasing some laboratory index (AFP) and immune functions (CD3^+^, CD4^+^, CD4^+^/CD8^+^, NK) and reducing some adverse effects (WBC decrease, gastrointestinal adverse effects). However, some results (6 months survival time, PLT decrease, HB decrease, liver damage, kidney damage, pain, fever and CD8^+^) cannot support a convincing conclusion because they cannot obtain the statistical difference. We should make the conclusion cautiously that this combined therapy can be applied as an auxiliary clinical treating method on non-surgical HCC treatment because many included publications’ low quality increases risk and bias and affects the reliability of study to some extent. So the traditional insect Chinese medicine and related preparation still need further standard, multicenter, double-blind randomized clinical trials (RCTs) to provide more clinical evidences in the future.

### Data Availability Statement

All data in this article are fully available without restriction.

## Electronic supplementary material

Supplementary Information
