# Supplementary material for: A systematic review and meta-analysis of traditional insect Chinese medicines combined chemotherapy for non-surgical hepatocellular carcinoma therapy
Source: Sci Rep. 2017 Jun 28;7:4355. doi: 10.1038/s41598-017-04351-y (PMC5489479; doi:10.1038/s41598-017-04351-y)
Supplement: Supplementary file 1 — Supplementary Information [file 41598_2017_4351_MOESM1_ESM.pdf]

Supplementary Information

A systematic review and meta-analysis of traditional insect Chinese medicines combined chemotherapy for non-surgical hepatocellular carcinoma therapy.

Zhaofeng Shi[1*], Tiebing Song[2*], Yi Wan[3*], Juan Xie[1], Yiquan Yan[1], Kekai Shi[5], Yongping Du[1], Lei Shang[4].

1. Department of Traditional Chinese Medicine, Xijing Hospital Affiliated to Forth Military Medical University, Xi'an 710032, China.

2. Department of Orthopaedics, Xi'an City Hospital of Traditional Chinese Medicine, Xi'an 710021, China.

3. Department of Health Services, the Public Health Faculty of Forth Military Medical University, Xi'an 710032, China.

4. Department of Health Statistics, the Public Health Faculty of Forth Military Medical University, Xi'an 710032, China.

5. Department of Engineering, Carnegie Mellon University, Pittsburgh, PA15213, the United States.

Correspondence and requests for materials should be addressed to L.S. (shanglei@fmmu.edu.cn)

*These authors contributed equally to this work.

1. The subgroup analysis for included evaluating indexes of the meta-analysis.

The subgroup analysis was performed by RevMan in the included evaluating indexes of the meta-analysis that have moderate or high heterogeneity (the result of I^2 was above 50%). The subgroup analysis was based on different separating criteria as the follows: (1) the drugs classification; (2) literature publication years; (3) experimental areas. If the results of subgroup analysis based on different separating criteria showed that the separating results of I^2 were remarkable lower than before, we would affirm the source of heterogeneity. The results of meta-analysis showed that white blood cell (WBC) decrease (I^2=85%), hemoglobin (HB) decrease (I^2=50%), gastrointestinal adverse reaction (I^2=62%), liver damage (I^2=92%), fever (I^2=72%), pain (I^2=80%) need to be conducted by the subgroup analysis. It is important to note that the immune functions (CD3+, CD4+, CD8+, CD4+/CD8+, NK) were not conducted the subgroup analysis because of the insufficient numbers of articles.

(1) The subgroup analysis of WBC decrease.

(a) The subgroup analysis based on drugs classification.

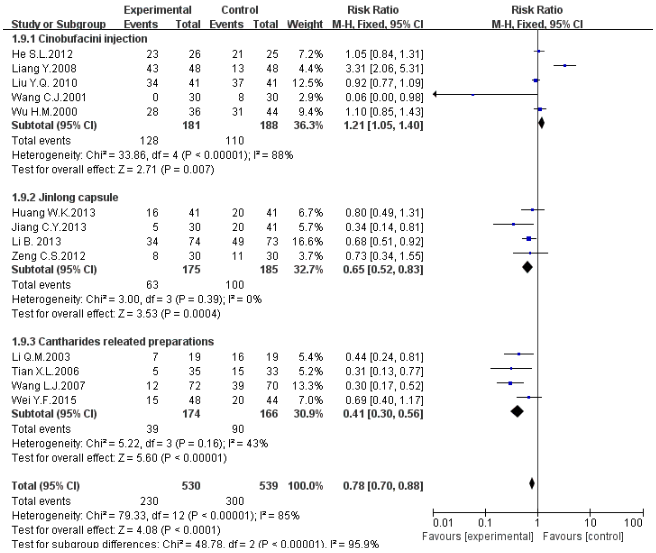

(b) The subgroup analysis based on experimental areas.

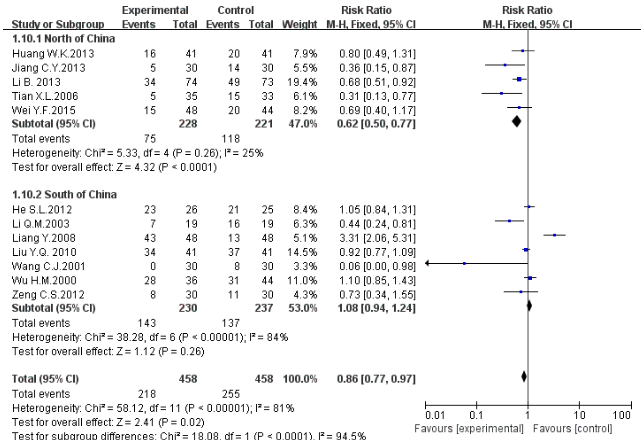

(c) The subgroup analysis based on literature publication years.

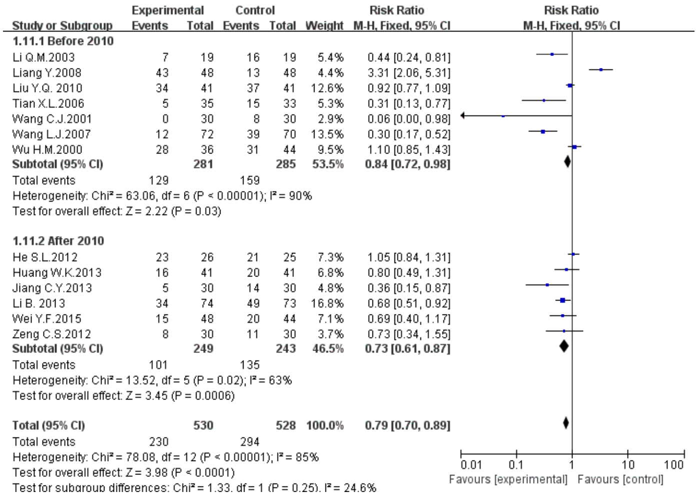

(2) The subgroup analysis of HB decrease.

(a) The subgroup analysis based on drugs classification.

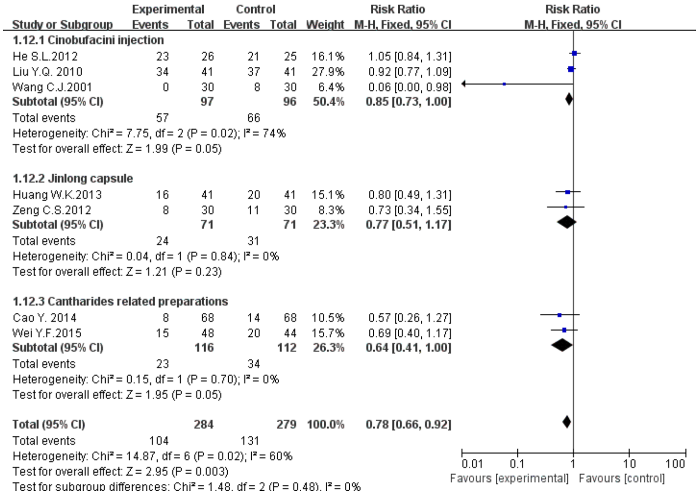

(b) The subgroup analysis based on experimental areas.

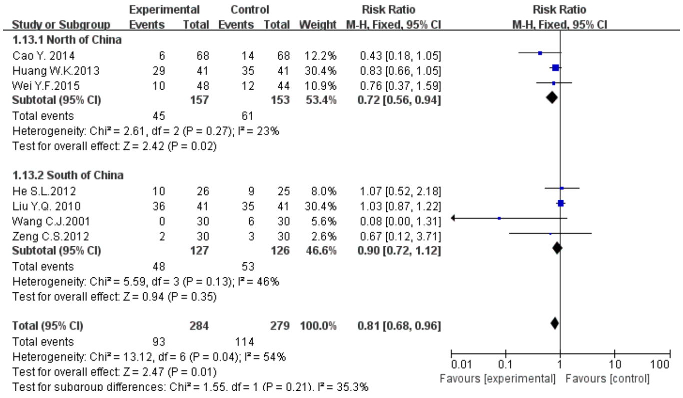

(c) The subgroup analysis based on literature publication years.

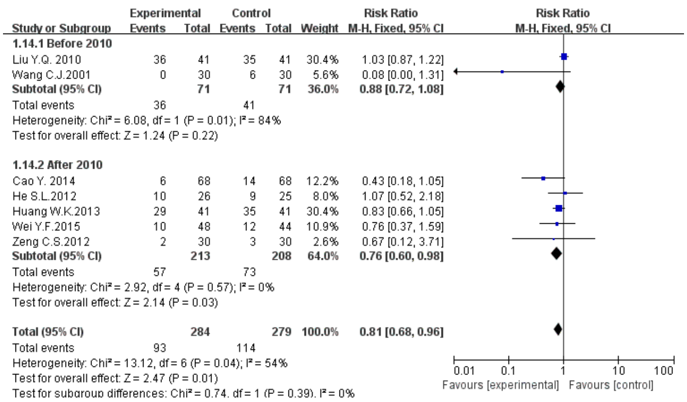

(3) The subgroup analysis of gastrointestinal adverse reaction.

(a) The subgroup analysis based on drugs classification.

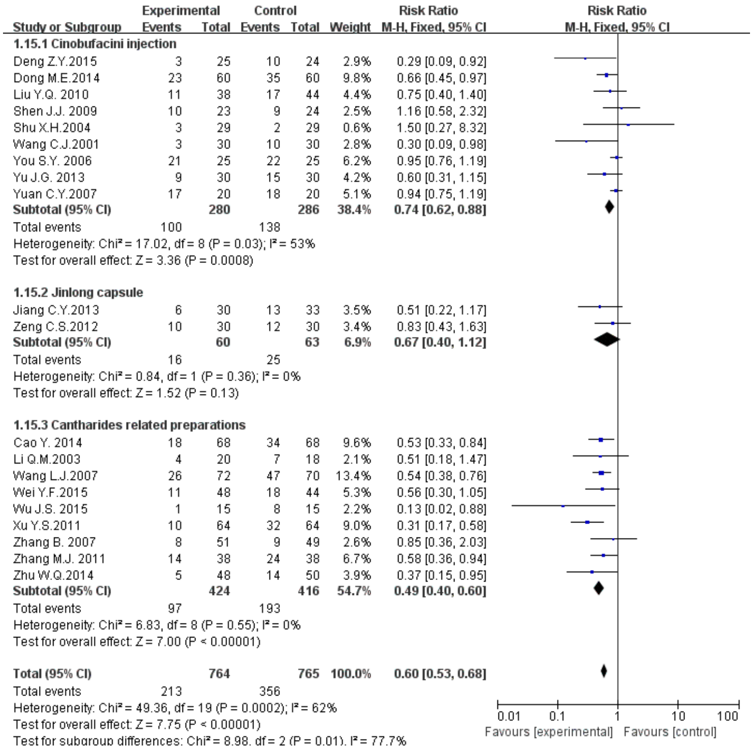

(b) The subgroup analysis based on experimental areas.

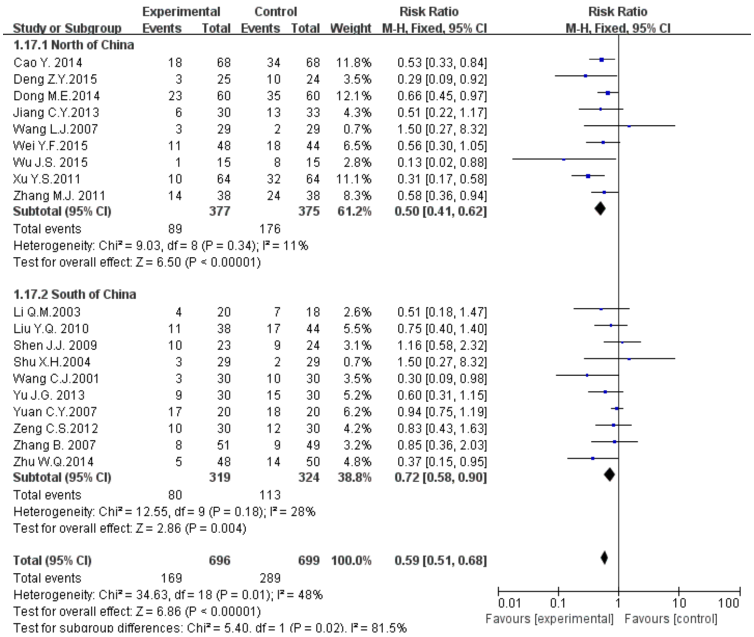

(c) **The subgroup analysis based on literature publication years.**

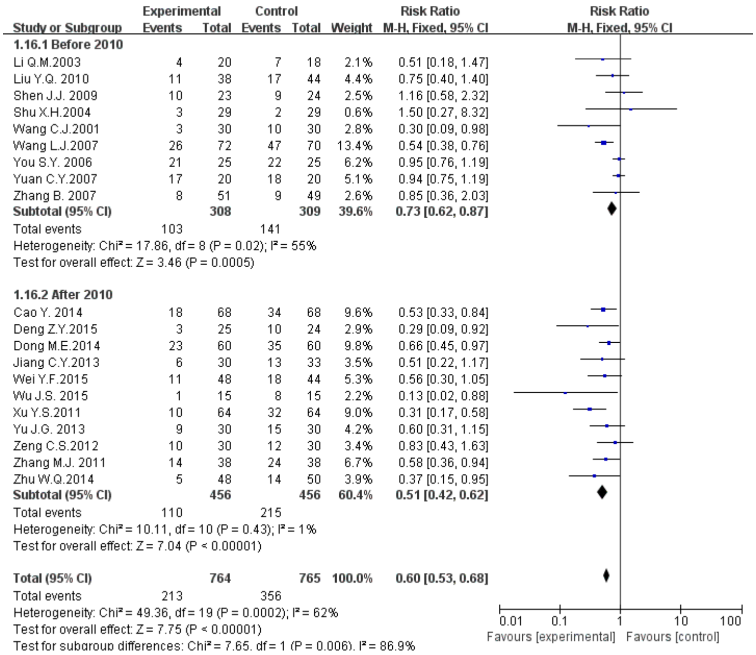

(4) The subgroup analysis of Liver damage.

(a) **The subgroup analysis based on drugs classification.**

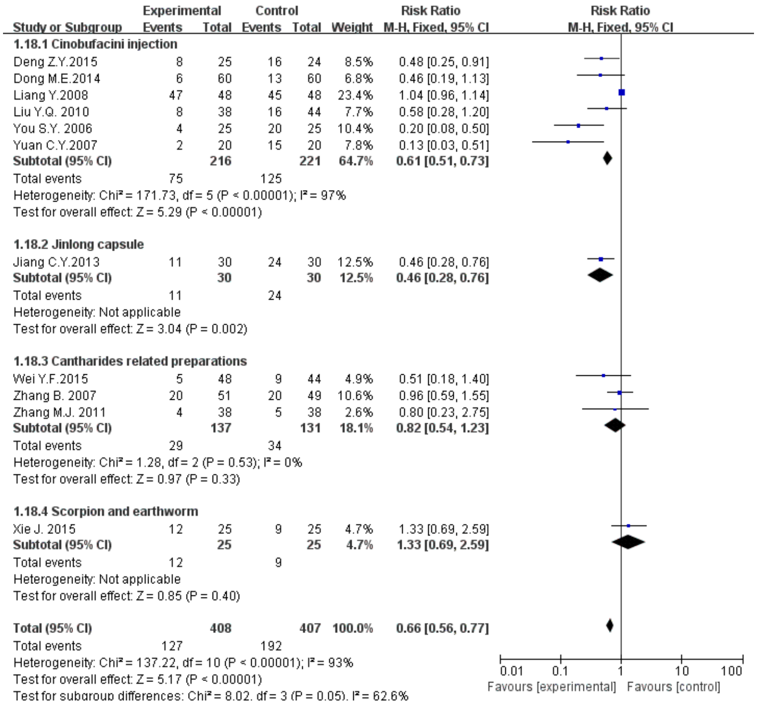

(b) The subgroup analysis based on experimental areas.

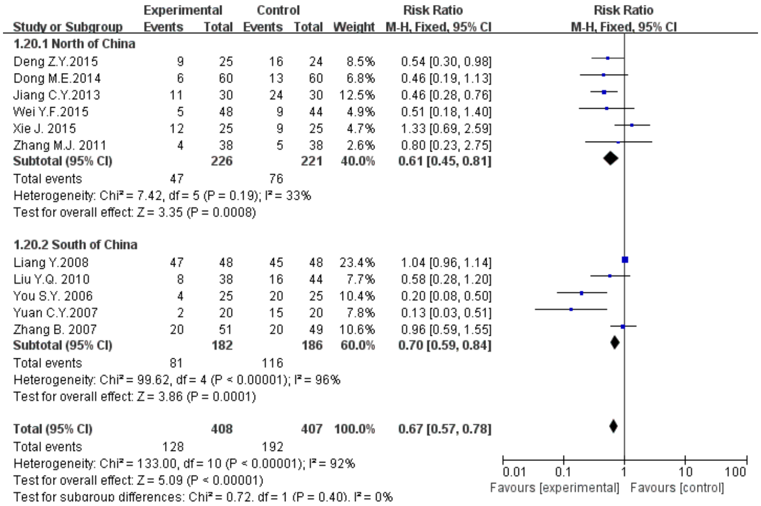

(c) The subgroup analysis based on literature publication years.

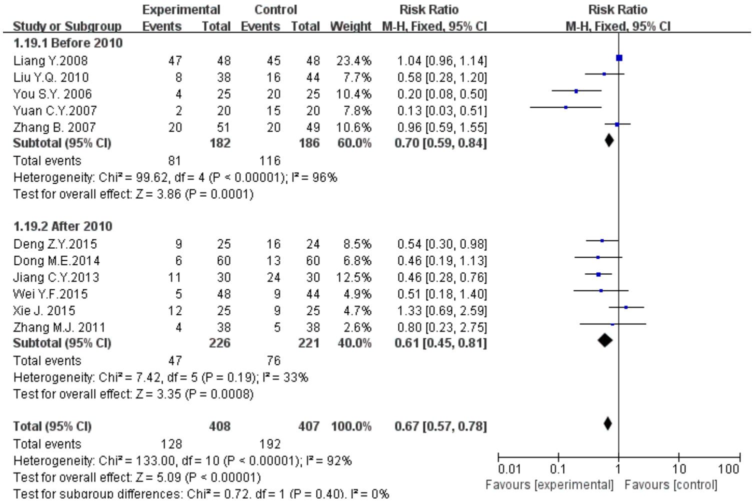

(5) The subgroup analysis of fever.

(a) The subgroup analysis based on drugs classification.

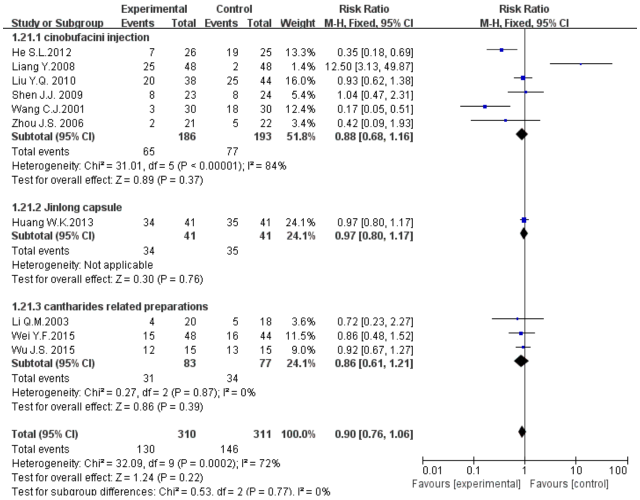

(b) The subgroup analysis based on experimental areas.

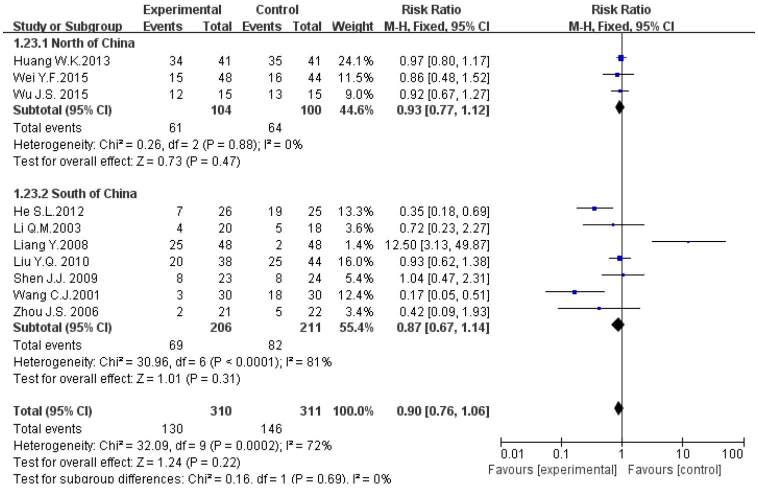

(c) The subgroup analysis based on literature publication years.

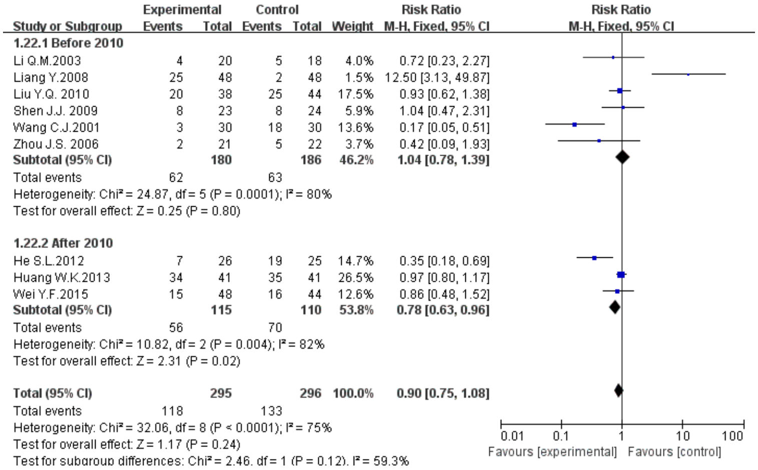

(6) The subgroup analysis of pain.

(a) The subgroup analysis based on drugs classification.

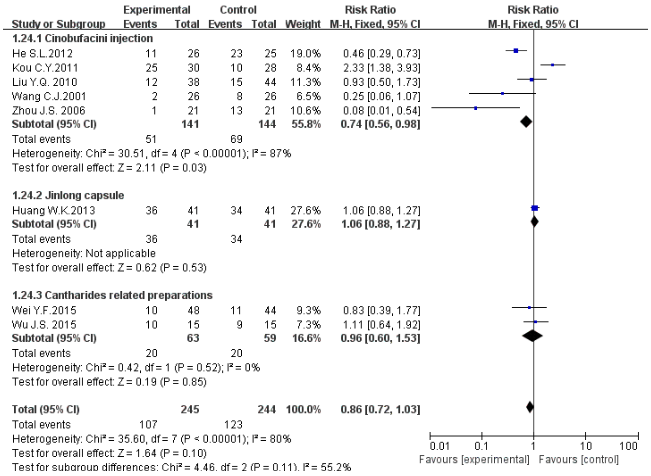

(b) The subgroup analysis based on experimental areas.

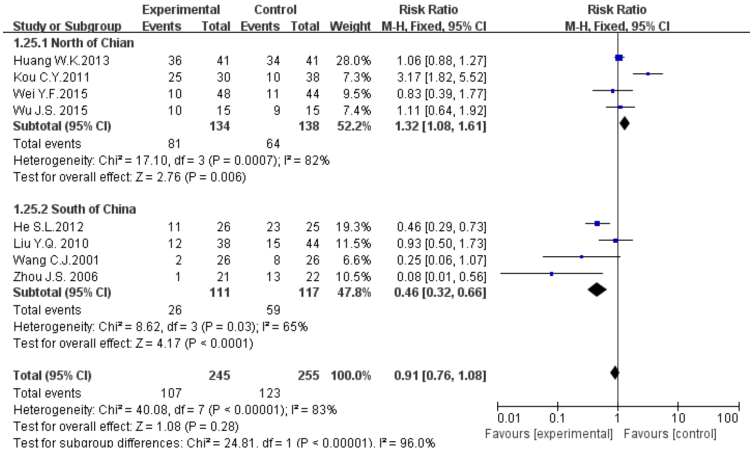

(c) The subgroup analysis based on literature publication years.

Study or Subgroup	Experimental Events	Experimental Total	Control Events	Control Total	Weight	Risk Ratio M-H, Fixed, 95% CI
**1.26.1 Before 2010**						
Liu Y.Q. 2010	12	38	15	44	11.3%	0.93 [0.50, 1.73]
Wang C.J.2001	2	26	8	26	6.5%	0.25 [0.06, 1.07]
Zhou J.S. 2006	1	21	13	22	10.3%	0.08 [0.01, 0.56]
**Subtotal (95% CI)**		**85**		**92**	**28.2%**	**0.46 [0.27, 0.78]**
Total events	15		36			
Heterogeneity: Chi² = 8.62, df = 2 (P = 0.01); I² = 77%						
Test for overall effect: Z = 2.90 (P = 0.004)						
**1.26.2 After 2010**						
He S.L.2012	11	26	23	25	19.1%	0.46 [0.29, 0.73]
Huang W.K.2013	36	41	34	41	27.7%	1.06 [0.88, 1.27]
Kou C.Y.2011	25	30	10	28	8.4%	2.33 [1.38, 3.93]
Wei Y.F.2015	10	48	11	44	9.3%	0.83 [0.39, 1.77]
Wu J.S. 2015	10	15	9	15	7.3%	1.11 [0.64, 1.92]
**Subtotal (95% CI)**		**160**		**153**	**71.8%**	**1.03 [0.86, 1.22]**
Total events	92		87			
Heterogeneity: Chi² = 21.52, df = 4 (P = 0.0002); I² = 81%						
Test for overall effect: Z = 0.27 (P = 0.78)						
**Total (95% CI)**		**245**		**245**	**100.0%**	**0.87 [0.73, 1.03]**
Total events	107		123			
Heterogeneity: Chi² = 35.23, df = 7 (P < 0.0001); I² = 80%						
Test for overall effect: Z = 1.61 (P = 0.11)						
Test for subgroup differences: Chi² = 8.02, df = 1 (P = 0.005), I² = 87.5%						

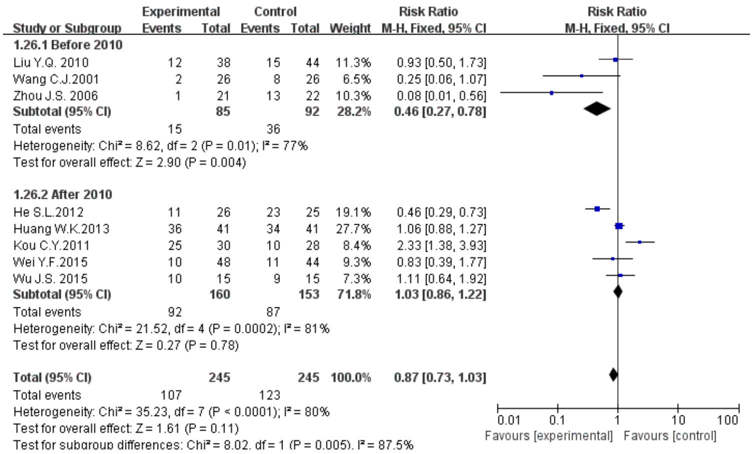

2. The images and detailed calculating information of Egger's and Begg's test by Stata.

Publication bias was assessed by Egger's test/Begg's test through the software named Stata (version 14.0, StataCrop LP, College Station, US). The *P* values lower than 0.05 were judged as statistically significant for the results, which represents that the study has publication bias. The results indicated that potential publication bias did exist

```
Note: data input format theta se_theta assumed.

Egger's test for small-study effects:
Regress standard normal deviate of intervention
effect estimate against its standard error

Number of studies =  46                          Root MSE      =   3.956

     Std_Eff        Coef.   Std. Err.       t    P>|t|     [95% Conf. Interval]

       slope     3.065244    1.19753     2.56   0.014     .6517819    5.478707
        bias    -7.324873   2.474271    -2.96   0.005    -12.31144   -2.338308

Test of H0: no small-study effects          P = 0.005
```

Egger's test (t=-2.96, P=0.005)

```
Note: data input format theta se_theta assumed.

Begg's test for small-study effects:
Rank correlation between standardized intervention effect and its standard error

  adj. Kendall's Score (P-Q) =     -179
          Std. Dev. of Score =   105.62
           Number of Studies =       46
                           z =    -1.69
                    Pr > |z| =    0.090
                           z =     1.69 (continuity corrected)
                    Pr > |z| =    0.092 (continuity corrected)
```

Begg's test (z=1.96, P=0.092)
